# Trends and disparities in deaths involving atherosclerotic cardiovascular disease and stroke-related conditions among U.S. adults, 1999–2025

**DOI:** 10.3389/fcvm.2026.1851946

**Published:** 2026-07-16

**Authors:** Muhammad Atif Mazhar, Abdal Ahmad, Vishan Das, Danyal Ahmad, Kaneez Fatima, Muhammad Mukhlis, Eshal Atif, Zubair Ahmed, Sadia Qazi

**Affiliations:** 1 Department of Anatomy, College of Medicine, Alfaisal University, Riyadh, Saudi Arabia; 2 Department of Medicine, Peshawar Medical College, Riphah International University, Peshawar, Pakistan; 3 Liaquat University of Medical & Health Sciences, Jamshoro, Pakistan; 4 Peshawar Institute of Cardiology, Peshawar, Pakistan; 5 CMH Institute of Medical Sciences, Multan, Pakistan; 6 Ayub Medical College, Abbottabad, Pakistan; 7 College of Medicine, Alfaisal University, Riyadh, Saudi Arabia; 8 Internal Medicine Department, Richmond University Medical Center, Staten Island, NY, United States

**Keywords:** atherosclerotic cardiovascular disease, cardiovascular epidemiology, health disparities, prevention, stroke mortality

## Abstract

**Background:**

ASCVD and stroke remain leading causes of death in the United States, sharing overlapping risk factors and clinical consequences. Long-term declines in vascular mortality have slowed while disparities persist. We evaluated national trends and disparities in mortality involving coexisting ASCVD- and stroke-related conditions among U.S. adults from 1999 to 2025.

**Methods:**

We performed a retrospective population-based study using the CDC WONDER Multiple Cause of Death database. Adults aged ≥25 years were included. Deaths were identified when prespecified ASCVD-related and stroke-related ICD-10 codes were both documented on the same death certificate, whether as underlying or contributing causes. The outcome represents a death-certificate-defined mortality phenotype rather than clinically adjudicated concurrent disease. Age-adjusted mortality rates (AAMRs) per 100,000 were calculated using the 2000 U.S. standard population. Trends were assessed overall and by sex, age group, race/ethnicity, census region, urbanization, and state. Urbanization analyses were restricted to 1999–2020. Sensitivity analyses included restriction to atherosclerotic-specific codes (I25.x) and exclusion of provisional 2025 data.

**Results:**

A total of 876,383 deaths were identified. Overall average AAMR was 15.01 per 100,000. Mortality declined from 26.82 in 1999 to 10.91 per 100,000 in 2025, with an AAPC of −3.53% (95% CI, −4.27 to −2.78; *p* < 0.001). After sustained declines through 2018, AAMR showed a borderline significant increase during 2018–2021 (APC: 6.06%; 95% CI, 0.01–12.48; *p* = 0.050), not replicated under stricter cause-of-death definitions, followed by renewed decline from 2021 to 2025 (APC: −2.85%; 95% CI, −4.58 to −1.09; *p* = 0.004). Mortality burden remained higher among men, older adults, Black individuals, residents of the South, and non-metropolitan populations. Adults aged 25–44 years showed a significant increase after 2015, though absolute rates remain low and this finding warrants cautious interpretation.

**Conclusion:**

Although mortality involving coexisting ASCVD- and stroke-related conditions declined substantially from 1999 to 2025, this progress was interrupted by a borderline reversal in the pandemic period and remained marked by persistent disparities. These findings support the need for stronger and more equitable prevention strategies, particularly for younger adults and high-burden populations and regions.

## GRAPHICAL ABSTRACT

**Figure:**
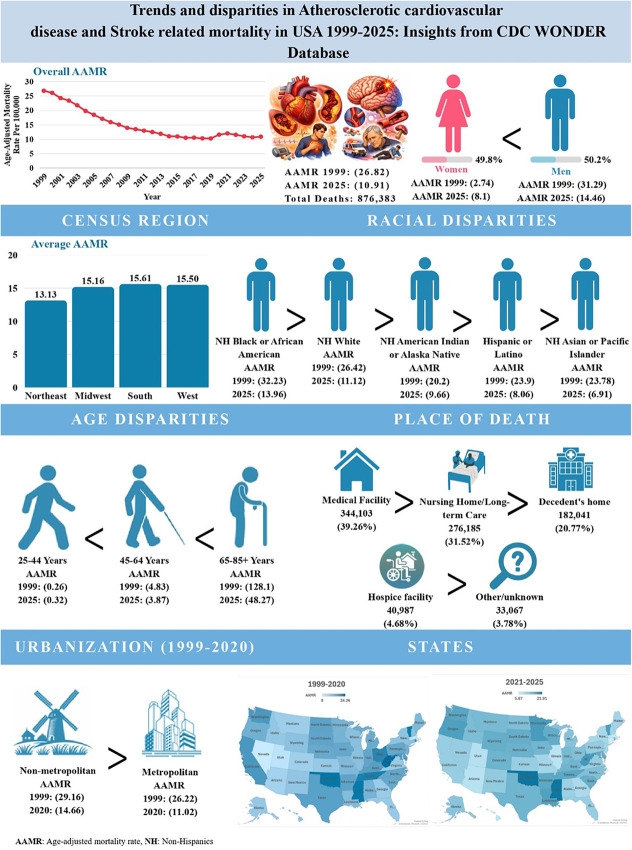

## 1 Introduction

Atherosclerotic cardiovascular disease (ASCVD) remains a leading cause of mortality in the United States despite major advances in prevention, risk-factor modification, and acute cardiovascular care (1). From the perspective of cardiovascular epidemiology and vascular medicine, recent national analyses suggest that the long-term decline in ASCVD mortality has slowed, with evidence of plateauing and renewed increases in atherosclerotic heart disease and broader cardiovascular mortality in recent years (1–3). Stroke likewise continues to impose a substantial public health burden in the United States, accounting for a major share of vascular mortality and affecting large numbers of individuals annually (4–6). Earlier reductions in stroke mortality have not been sustained uniformly across the population, and recent studies have shown unfavorable reversals in stroke-related death rates, particularly among younger and middle-aged adults (5, 6). Taken together, these findings suggest that progress in reducing ASCVD- and stroke-related mortality has become less consistent over time and that renewed emphasis on prevention is needed across the spectrum of vascular disease (1–6).

These unfavorable trends are accompanied by substantial disparities across demographic and geographic groups. Mortality related to ASCVD and stroke varies importantly by sex, race and ethnicity, and place of residence, with higher burdens reported among men, Black and American Indian/Alaska Native populations, and residents of non-metropolitan communities (4, 7). Such patterns underscore the importance of examining vascular mortality through the lens of public health, equity, and disparities. In addition, ASCVD and stroke are closely linked within a shared vascular risk framework. Cardiovascular dysfunction, including heart failure and related vascular abnormalities, is associated with increased stroke risk, while stroke itself may adversely affect cardiac structure and function through hemodynamic, inflammatory, and neurohumoral pathways (8). This bidirectional relationship highlights the clinical and pathophysiologic overlap between cardiovascular and cerebrovascular disease and supports an integrated vascular medicine approach to risk assessment and prevention (9).

At the population level, however, death-certificate data do not directly establish clinically adjudicated concurrent disease in an individual decedent. Rather, they capture mortality involving coexisting ASCVD- and stroke-related conditions as documented on the same death certificate, whether as underlying or contributing causes of death. Accordingly, analyses based on the Multiple Cause of Death database are best interpreted as evaluations of a death-certificate phenotype of coexisting ASCVD- and stroke-related conditions, rather than confirmed simultaneous clinical diagnoses. This distinction is important because death-certificate coding may reflect varying combinations of active disease, antecedent vascular injury, sequelae, or certifier attribution.

Against this background, a contemporary national evaluation of mortality involving coexisting ASCVD- and stroke-related conditions from 1999 to 2025 is warranted. Such an analysis may better define temporal trends in the documentation of coexisting ASCVD- and stroke-related conditions on death certificates, characterize persistent demographic and geographic disparities in this mortality phenotype, and inform more equitable prevention and policy strategies aimed at improving cardiovascular and cerebrovascular outcomes in the United States.

## 2 Methodology

### 2.1 Study design, population, and case identification

Mortality data were obtained from the Centers for Disease Control and Prevention Wide-Ranging Online Data for Epidemiologic Research (CDC WONDER) Multiple Cause of Death database, which contains publicly available, de-identified death certificate records from all 50 U.S. states and the District of Columbia (10, 11). We conducted a retrospective, population-based analysis of deaths recorded between 1999 and 2025.

The analysis was restricted to adults aged ≥25 years because mortality involving vascular conditions is uncommon at younger ages and less stable for population-level trend analyses. The primary outcome was mortality involving coexisting ASCVD- and stroke-related conditions, defined as death records in which prespecified ASCVD-related and stroke-related ICD-10 code groups were both documented on the same death certificate, whether as underlying or contributing causes of death. Deaths were identified using the International Classification of Diseases, Tenth Revision (ICD-10) coding framework (12).

Stroke-related conditions were identified using ICD-10 codes; I60 (subarachnoid hemorrhage), I61 (intracerebral hemorrhage), I63 (cerebral infarction), I64 (stroke, not specified as hemorrhage or infarction), I69.0 (sequelae of subarachnoid hemorrhage), I69.1 (sequelae of intracerebral hemorrhage), I69.3 (sequelae of cerebral infarction), and I69.4 (sequelae of stroke, not specified as hemorrhage or infarction). ASCVD-related conditions were identified using ICD-10 codes I25.0 (atherosclerotic cardiovascular disease, so described), I25.1 (atherosclerotic heart disease), I25.2 (old myocardial infarction), I25.5 (ischemic cardiomyopathy), I25.6 (silent myocardial ischemia), I25.8 (other forms of chronic ischemic heart disease), I25.9 (chronic ischemic heart disease, unspecified), I51.3 (intracardiac thrombosis, not elsewhere classified), I51.7 (cardiomegaly), I51.9 (heart disease, unspecified), I87.8 (other specified disorders of veins), I87.9 (disorder of vein, unspecified), I99 (other and unspecified disorders of circulatory system), and R93.1 (abnormal findings on diagnostic imaging of heart and coronary circulation). These code groups were selected to preserve comparability with prior U.S. mortality studies using identical code sets (4, 13, 14), including a recently published national analysis of obesity and ASCVD mortality that employed the same broad circulatory code selection and explicitly acknowledged the same non-specific categories (13).

Because the primary code set includes several broad or non-specific circulatory categories, the outcome should be interpreted as a death-certificate-defined mortality phenotype rather than clinically adjudicated simultaneous disease. In particular, inclusion of I51.3, I51.7, I51.9, I87.8, I87.9, I99, and R93.1 may capture overlapping or non-specific circulatory pathology and could contribute to miscoding, selection bias, or overestimation of atherosclerotic mortality. To directly assess whether these broader codes materially influenced the observed temporal patterns, we conducted three pre-specified sensitivity analyses: (1) restriction of the ASCVD definition to atherosclerotic-specific codes only (I25), retaining the same stroke code group (I60, I61, I63, I64, I69.0, I69.1, I69.3 and I69.4), with Joinpoint trend analysis applied to this restricted dataset across the full 1999–2025 period; (2) the same I25.x-restricted analysis stratified by sex; and (3) replication of the primary analysis excluding provisional 2025 data, using the confirmed 1999–2024 series, to assess whether inclusion of the terminal provisional year influenced the trend estimates. Results of these sensitivity analyses are reported separately.

### 2.2 Data extraction and variables

For each eligible death record, we extracted year of death, sex, age group, race and ethnicity, census region, state, urbanization category, and place of death. Total deaths, crude mortality rates per 100,000 population, and age-adjusted mortality rates (AAMRs) were evaluated across these strata.

Sex in death certificate records reflects sex as recorded on the certificate and generally corresponds to sex assigned at birth. Separate data on gender identity or gender-diverse populations are not available within CDC WONDER. Race and ethnicity were classified according to CDC WONDER categories as non-Hispanic (NH) White, NH Black or African American, American Indian or Alaska Native, Asian or Pacific Islander, and Hispanic or Latino. Geographic regions were grouped according to U.S. Census Bureau definitions as Northeast, Midwest, South, and West (15). Urban-rural classification was based on the 2013 National Center for Health Statistics Urban-Rural Classification Scheme (16). To preserve a consistent long-term series, urbanization-stratified analyses were limited to the archived 1999–2020 data.

Place of death was categorized as medical facility inpatient, medical facility outpatient or emergency room, nursing home or long-term care facility, hospice facility, decedent's home, dead on arrival, other, unknown place of death, and medical facility status unknown. As with other CDC WONDER analyses, race and ethnicity on death certificates should be interpreted cautiously because they are proxy-reported rather than self-identified and may be subject to misclassification. This concern is especially relevant for American Indian and Alaska Native populations and is considered in the interpretation of results (17–19).

Data were extracted from CDC WONDER on 2 March 2026. Two separate queries were conducted and subsequently merged: the first covered 1999–2020 using the standard Multiple Cause of Death file, and the second covered 2021–2025, with data for 2024 representing final confirmed records and 2025 representing provisional data available at the time of extraction. The merged dataset spanned 1999–2025. Query parameters included: geographic scope, all 50 U.S. states and the District of Columbia; age restriction, 25 years and older; ICD-10 multiple cause of death codes as specified above; grouping variables, year, sex, age group, race and ethnicity, census region, urbanization category, state, and place of death; and standard population, 2000 U.S. Standard Population for age adjustment. No records were suppressed or flagged as unreliable in the extracted dataset, as all subgroup strata queried returned death counts of 20 or more in every calendar year (20, 21).

### 2.3 Data analysis and ethical considerations

AAMRs per 100,000 population were calculated overall and across demographic and geographic subgroups, directly standardized to the 2000 U.S. standard population (22), and corresponding 95% confidence intervals (CIs) were calculated for mortality estimates. These rates represent the population burden of mortality involving coexisting ASCVD and stroke-related conditions as documented on death certificates, rather than the incidence or prevalence of clinically confirmed concurrent disease.

Temporal trends in AAMRs were evaluated using the Joinpoint Regression Program from the National Cancer Institute (23). Log-linear models were used to identify statistically significant changes in mortality trends over time. Annual percent change (APC) was estimated for each identified time segment, and average annual percent change (AAPC) was used to summarize the overall trend across the full study period (24). All APC and AAPC estimates were reported with 95% CIs, and statistical significance was defined as a two-sided *p* value < 0.05 (25, 26). Subgroup analyses were performed by sex, age group, race and ethnicity, census region, urbanization, and state. State-level analyses were summarized using average AAMRs and percentile-based ranking to identify the highest- and lowest-burden states.

Four sensitivity analyses were pre-specified to test the robustness of the primary findings. First (S1), the ASCVD code group was restricted to atherosclerotic-specific codes (I25.x) co-listed with stroke codes (I60, I61, I63, I64, I69.0, I69.1, I69.3, and I69.4), excluding all non-specific circulatory codes, and Joinpoint trend analysis was rerun on this restricted dataset across the full 1999–2025 period. Second (S2), this I25.x-restricted analysis was repeated with sex stratification separately for females (S2a) and males (S2b) to assess whether sex-specific temporal patterns were similarly robust. Third (S3), the primary analysis was replicated using the confirmed 1999–2024 data series, excluding provisional 2025 data, to evaluate whether the terminal year influenced trend estimates; this confirmed-data analysis was also repeated with sex stratification (S3a: females; S3b: males). Fourth (S4), four alternative cause-of-death definitions were applied to assess sensitivity to death-certificate coding: ASCVD as the underlying cause of death (UCD ASCVD); stroke as the underlying cause of death (UCD Stroke); ASCVD as the underlying cause with stroke as a contributing cause (UCD ASCVD-with-Stroke); and stroke as the underlying cause with ASCVD as a contributing cause (UCD Stroke-with-ASCVD). These analyses were intended to assess whether the observed temporal patterns were sensitive to different operational definitions of mortality and to the inclusion of provisional data. As no suppressed or unreliable cells were present in the extracted data, all annual observations were included in Joinpoint models without imputation or exclusion. A STROBE-compliant case ascertainment flow is provided in Supplementary Figure S1.

Because the study used publicly available, de-identified mortality data derived from existing death certificate records, institutional review board approval was not required. The study was conducted and reported in accordance with the Strengthening the Reporting of Observational Studies in Epidemiology (STROBE) statement (27).

## 3 Results

**Figure 1:**
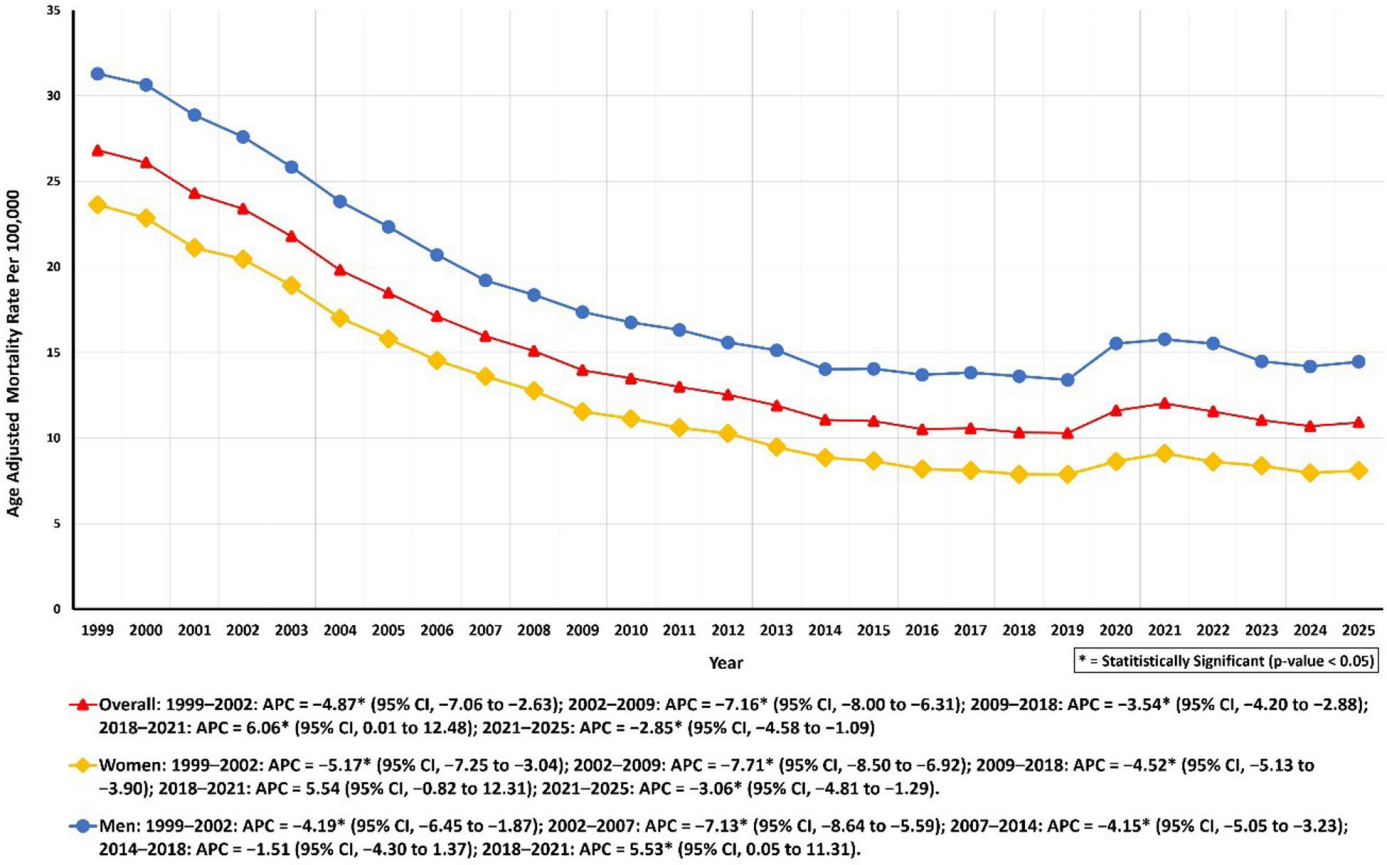
Overall and sex-stratified age-adjusted mortality rates (per 100,000) for mortality involving coexisting ASCVD- and stroke-related conditions among U.S. adults aged ≥25 years, 1999–2025.

Between 1999 and 2025, a total of 876,383 deaths involving coexisting ASCVD- and stroke-related conditions were identified among U.S. adults aged 25 years and older following the case ascertainment procedure described in Supplementary Figure S1, with an average age-adjusted mortality rate (AAMR) of 15.01 per 100,000. Overall, the AAMR declined from 26.82 (95% CI: 26.57–27.06) in 1999 to 10.91 (95% CI: 10.79–11.03) in 2025, corresponding to a significant overall AAPC of −3.53 (95% CI: −4.27 to −2.78, *p* < 0.001). Joinpoint analysis showed that this decline was not uniform over time. Mortality decreased significantly from 1999 to 2002 (APC: −4.87; 95% CI: −7.06 to −2.63, *p* < 0.001), followed by a steeper decline from 2002 to 2009 (APC: −7.16; 95% CI: −8.00 to −6.31, *p* < 0.001). A further significant decline was observed from 2009 to 2018 (APC: −3.54; 95% CI: −4.20 to −2.88, *p* < 0.001). This long-term downward trend was interrupted by an increase from 2018 to 2021 (APC: 6.06; 95% CI: 0.01–12.48, *p* = 0.050), which reached the threshold of statistical significance but should be interpreted with caution given the confidence interval lower bound of 0.01 and the attenuation of this signal under stricter code definitions, as described in Section 3.8. Mortality declined again from 2021 to 2025 (APC: −2.85; 95% CI: −4.58 to −1.09, *p* = 0.004) (Table 1, Figure 1, Supplementary Table S1).

**Table 1:** Annual percent change (APC) and average annual percent change (AAPC) of mortality involving coexisting ASCVD- and stroke-related conditions among adults aged ≥25 years in the United States, 1999–2025.

Year	APC (95% CI)	*p*-value	AAPC (95% CI)	*p*-value
Overall				
1999–2002	−4.87% (−7.06% to −2.63%)	0.001		
2002–2009	−7.16% (−8.00% to −6.31%)	<0.001		
2009–2018	−3.54% (−4.20% to −2.88%)	<0.001		
2018–2021	6.06% (0.01% to 12.48%)	0.050		
2021–2025	−2.85% (−4.58% to −1.09%)	0.004		
1999–2025			−3.53% (−4.27% to −2.78%)	<0.001
Sex				
Women				
1999–2002	−5.17% (−7.25% to −3.04%)	0.001		
2002–2009	−7.71% (−8.50% to −6.92%)	<0.001		
2009–2018	−4.52% (−5.13% to −3.90%)	<0.001		
2018–2021	5.54% (−0.82% to 12.31%)	0.084		
2021–2025	−3.06% (−4.81% to −1.29%)	0.003		
1999–2025			−4.14% (−4.90% to −3.38%)	<0.001
Men				
1999–2002	−4.19% (−6.45% to −1.87%)	0.003		
2002–2007	−7.13% (−8.64% to −5.59%)	<0.001		
2007–2014	−4.15% (−5.05% to −3.23%)	<0.001		
2014–2018	−1.51% (−4.30% to 1.37%)	0.267		
2018–2021	5.53% (0.05% to 11.31%)	0.048		
1999–2025			−3.05% (−3.85% to −2.25%)	<0.001
Race and ethnicity				
NH American Indian or Alaska Native				
1999–2014	−3.81% (−5.01% to −2.58%)	<0.001		
2014–2025	−0.76% (−2.53% to 1.05%)	0.391		
1999–2025			−2.53% (−3.50% to −1.55%)	<0.001
NH Asian or Pacific Islander				
1999–2015	−6.80% (−7.44% to −6.15%)	<0.001		
2015–2025	−0.64% (−1.89% to 0.64%)	0.310		
1999–2025			−4.47% (−5.06% to −3.89%)	<0.001
NH Black or African American				
1999–2016	−5.69% (−6.15% to −5.24%)	<0.001		
2016–2021	3.84% (−0.73% to 8.62%)	0.096		
2021–2025	−2.70% (−6.77% to 1.55%)	0.197		
1999–2025			−3.47% (−4.49% to −2.44%)	<0.001
NH White				
1999–2002	−4.83% (−6.65% to −2.98%)	0.001		
2002–2007	−7.61% (−8.91% to −6.28%)	<0.001		
2007–2014	−4.59% (−5.40% to −3.78%)	<0.001		
2014–2018	−2.14% (−4.76% to 0.54%)	0.104		
2018–2021	5.54% (0.08% to 11.30%)	0.047		
1999–2025			−3.38% (−4.13% to −2.63%)	<0.001
Hispanic or Latino				
1999–2015	−6.21% (−6.75% to −5.66%)	<0.001		
2015–2025	−0.43% (−1.50% to 0.65%)	0.420		
1999–2025			−4.03% (−4.52% to −3.53%)	<0.001
Age group				
25–44 years				
1999–2015	−0.23% (−1.38% to 0.93%)	0.686		
2015–2025	4.45% (2.39% to 6.55%)	<0.001		
1999–2025			1.55% (0.55% to 2.55%)	0.002
45–64 years				
1999–2008	−4.57% (−5.25% to −3.88%)	<0.001		
2008–2017	−1.04% (−1.93% to −0.15%)	0.025		
2017–2021	8.11% (4.24% to 12.13%)	<0.001		
2021–2025	−0.78% (−3.13% to 1.62%)	0.497		
1999–2025			−0.90% (−1.61% to −0.18%)	0.014
65–85 + years				
1999–2002	−4.90% (−7.20% to −2.54%)	0.001		
2002–2009	−7.39% (−8.26% to −6.50%)	<0.001		
2009–2018	−3.92% (−4.59% to −3.23%)	<0.001		
2018–2021	5.63% (−0.89% to 12.58%)	0.086		
1999–2025			−3.83% (−4.62% to −3.03%)	<0.001
Urbanization (1999–2020)				
Metropolitan				
1999–2009	−6.84% (−7.27% to −6.40%)	<0.001		
2009–2018	−3.86% (−4.60% to −3.12%)	<0.001		
2018–2020	7.31% (0.00% to 15.16%)	0.050		
1999–2020			−4.29% (−4.98% to −3.61%)	<0.001
Non-metropolitan				
1999–2003	−3.95% (−5.03% to −2.86%)	<0.001		
2003–2006	−7.66% (−11.27% to −3.90%)	0.002		
2006–2014	−4.65% (−5.23% to −4.08%)	<0.001		
2014–2018	−1.15% (−3.50% to 1.25%)	0.298		
2018–2020	6.47% (1.75% to 11.41%)	0.013		
1999–2020			−3.29% (−4.03% to −2.54%)	<0.001
Census region				
Northeast				
1999–2008	−7.29% (−7.93% to −6.64%)	<0.001		
2008–2017	−4.51% (−5.49% to −3.52%)	<0.001		
2017–2021	2.66% (−2.09% to 7.63%)	0.257		
2021–2025	−4.45% (−7.34% to −1.47%)	0.006		
1999–2025			−4.41% (−5.27% to −3.55%)	<0.001
Midwest				
1999–2012	−6.05% (−6.28% to −5.81%)	<0.001		
2012–2018	−2.55% (−3.77% to −1.32%)	<0.001		
2018–2021	5.39% (−0.41% to 11.51%)	0.067		
2021–2025	−2.54% (−4.19% to −0.87%)	0.006		
1999–2025			−3.45% (−4.13% to −2.76%)	<0.001
South				
1999–2002	−4.00% (−6.27% to −1.67%)	0.004		
2002–2007	−7.40% (−8.94% to −5.84%)	<0.001		
2007–2014	−4.72% (−5.66% to −3.78%)	<0.001		
2014–2018	−1.50% (−4.35% to 1.43%)	0.276		
2018–2021	6.47% (0.69% to 12.59%)	0.031		
2021–2025	−2.28% (−3.90% to −0.64%)	0.011		
1999–2025			−3.07% (−3.89% to −2.24%)	<0.001
West				
1999–2002	−4.33% (−7.02% to −1.57%)	0.005		
2002–2009	−7.72% (−8.70% to −6.72%)	<0.001		
2009–2018	−3.95% (−4.70% to −3.19%)	<0.001		
2018–2021	4.67% (−2.59% to 12.46%)	0.193		
2021–2025	−2.93% (−5.03% to −0.78%)	0.012		
1999–2025			−3.92% (−4.81% to −3.02%)	<0.001

APC, annual percent change; AAPC, average annual percent change; CI, confidence interval; NH, non-Hispanic; UCD, underlying cause of death. Urbanization analyses cover 1999–2020 only, consistent with the archived CDC WONDER Multiple Cause of Death file for that period. AAPC rows summarize the overall trend across the full period shown; APC rows report individual Joinpoint-identified segments. *p* = 0.000 in original Joinpoint output indicates *p* < 0.001; all such values are reported as <0.001 throughout.

### 3.1 Sex-stratified trends

Sex-stratified analyses showed a persistently higher mortality burden among males than females. Males accounted for 439,126 deaths and had the higher average AAMR (18.61), whereas females accounted for 437,257 deaths with a lower average AAMR of 12.37. Among females, the AAMR decreased from 23.64 (95% CI: 23.35–23.93) in 1999 to 8.10 (95% CI: 7.96–8.24) in 2025, yielding a significant overall AAPC of −4.14 (95% CI: −4.90 to −3.38, *p* < 0.001). The decline in females was significant from 1999 to 2002, 2002 to 2009, and 2009 to 2018, followed by a non-significant increase between 2018 and 2021 and a renewed decline from 2021 to 2025. Among males, the AAMR decreased from 31.29 (95% CI: 30.85–31.73) in 1999 to 14.46 (95% CI: 14.24–14.68) in 2025, corresponding to an AAPC of −3.05 (95% CI: −3.85 to −2.25, *p* < 0.001). In males, mortality declined significantly during 1999–2002, 2002–2007, and 2007–2014, remained relatively stable during 2014–2018, and then increased significantly during 2018–2021 (APC: 5.53; 95% CI: 0.05–11.31, *p* = 0.048). These patterns indicate that although both sexes experienced long-term mortality improvement, the decline was steeper in females, while males maintained consistently higher mortality rates throughout the study period (Figure 1, Table 1, Supplementary Table S1).

### 3.2 Age-stratified trends

**Figure 2:**
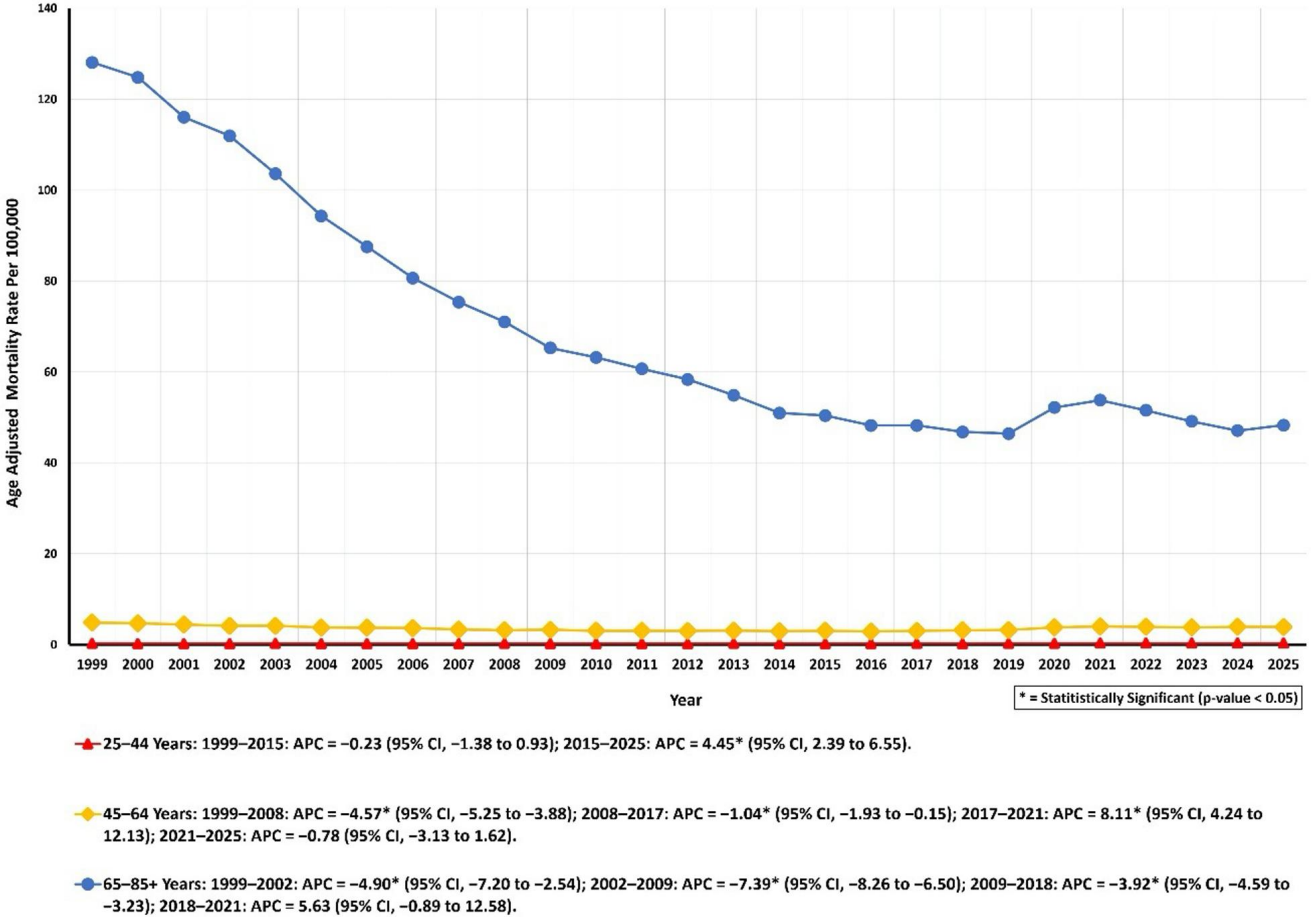
Age-stratified age-adjusted mortality rates per 100,000 for mortality involving coexisting ASCVD- and stroke-related conditions among U.S. adults aged ≥25 years, 1999–2025.

A marked age gradient was observed in mortality burden. Adults aged 65–85 + years had the highest average AAMR (69.95), followed by those aged 45–64 years (3.58), while adults aged 25–44 years had the lowest average AAMR (0.24). In the 25–44-year age group, the AAMR increased from 0.26 (95% CI: 0.23–0.30) in 1999 to 0.32 (95% CI: 0.28–0.36) in 2025, corresponding to a significant AAPC of 1.55 (95% CI: 0.55 to 2.55, *p* = 0.002). Although this increase is statistically significant, the absolute AAMR values in this age group are small (0.26 to 0.32 per 100,000), and annual death counts in this subgroup are low; therefore, this finding should be interpreted as a directional signal warranting surveillance rather than a stable quantitative estimate. Rates in this younger group remained relatively stable through 2015, but increased significantly thereafter from 2015 to 2025 (APC: 4.45; 95% CI: 2.39–6.55, *p* < 0.001). In the 45–64-year age group, the AAMR declined from 4.83 (95% CI: 4.65–5.00) in 1999 to 3.87 (95% CI: 3.74–4.00) in 2025, with an overall AAPC of −0.90 (95% CI: −1.61 to −0.18, *p* = 0.014). This group experienced significant declines during 1999–2008 and 2008–2017, followed by a sharp increase from 2017 to 2021 (APC: 8.11; 95% CI: 4.24–12.13, *p* < 0.001), and then a non-significant decline through 2025. In contrast, adults aged 65–85 + years showed a substantial reduction in mortality, with AAMRs declining from 128.10 (95% CI: 126.91–129.30) in 1999 to 48.27 (95% CI: 47.69–48.85) in 2025, corresponding to an AAPC of −3.83 (95% CI: −4.62 to −3.03, *p* < 0.001). Significant reductions were observed during 1999–2002, 2002–2009, and 2009–2018, followed by a non-significant increase during 2018–2021. Collectively, these findings indicate that the overall decline in mortality was driven primarily by improvements in older adults, whereas younger adults experienced worsening long-term trends (Figure 2, Table 1, Supplementary Table S2).

### 3.3 Race and ethnicity

**Figure 3:**
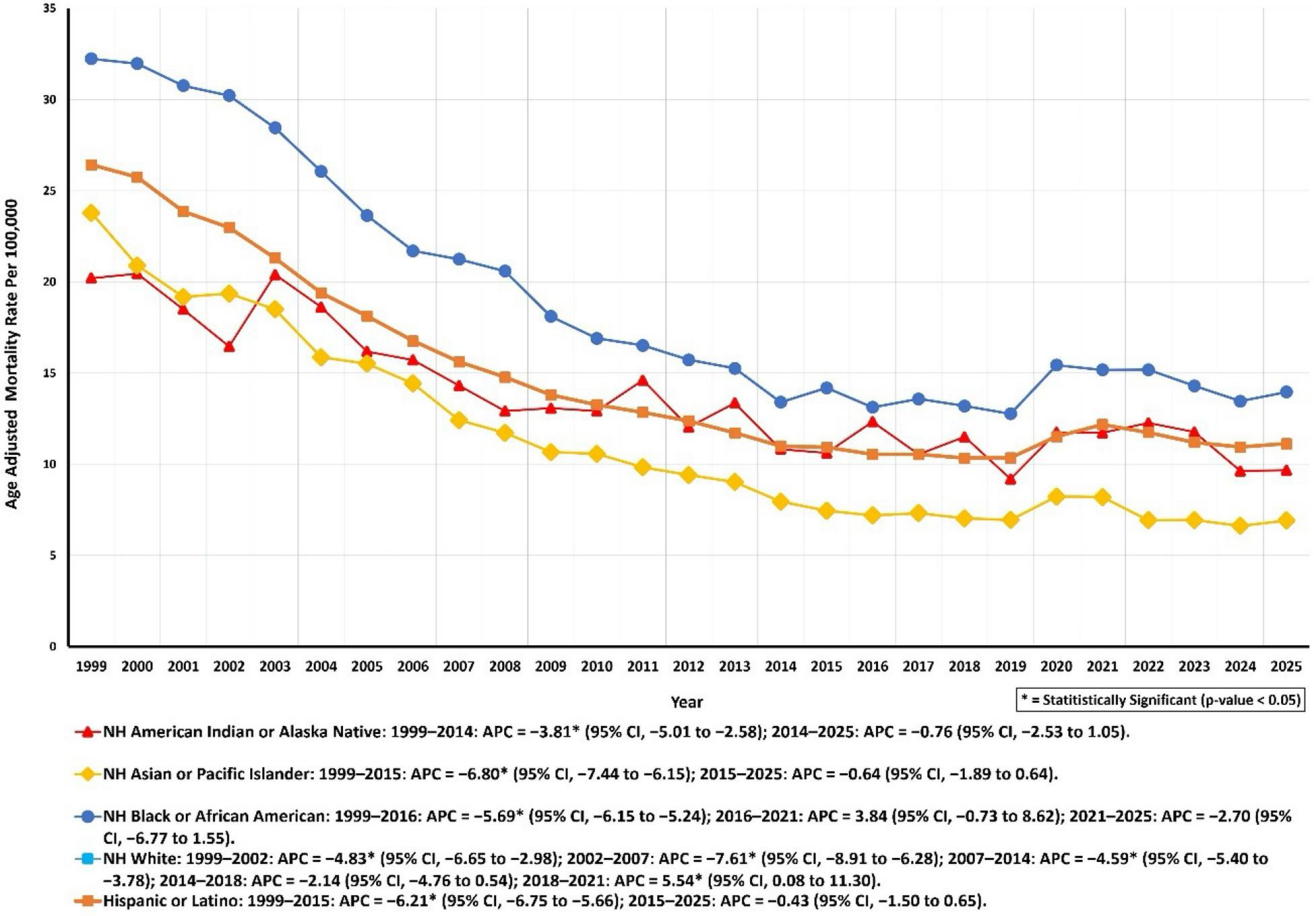
Race- and ethnicity-stratified age-adjusted mortality rates (per 100,000) for mortality involving coexisting ASCVD- and stroke-related conditions among U.S. adults aged ≥25 years, 1999–2025.

Substantial racial and ethnic disparities were evident throughout the study period. Black or African American adults had the highest average AAMR (19.15), followed by White adults (14.86), American Indian or Alaska Native adults (13.75), Hispanic or Latino adults (12.69), and Asian or Pacific Islander adults (11.43). Among Hispanic or Latino adults, AAMR declined from 23.90 (95% CI: 22.76–25.04) in 1999 to 8.06 (95% CI: 7.72–8.41) in 2025, with an overall AAPC of −4.03 (95% CI: −4.52 to −3.53, *p* < 0.001). In American Indian or Alaska Native adults, AAMR decreased from 20.20 (95% CI: 16.52–23.88) to 9.66 (95% CI: 8.25–11.25), corresponding to an AAPC of −2.53 (95% CI: −3.50 to −1.55, *p* < 0.001), see Supplementary Table S3. The wide confidence intervals around the American Indian or Alaska Native AAMR estimates reflect the smaller death counts in this group and should be interpreted with appropriate caution; annual death counts for this subgroup are provided in the Supplementary Table S4. Among Asian or Pacific Islander adults, AAMR fell from 23.78 (95% CI: 22.10–25.47) to 6.91 (95% CI: 6.50–7.33), yielding the steepest overall decline across racial and ethnic groups (AAPC: −4.47; 95% CI: −5.06 to −3.89, *p* < 0.001). In Black adults, the AAMR declined from 32.23 (95% CI: 31.28–33.17) in 1999 to 13.96 (95% CI: 13.52–14.42) in 2025, with an AAPC of −3.47 (95% CI: −4.49 to −2.44, *p* < 0.001), although this decline slowed and became non-significant after 2016. White adults also showed a significant long-term decline, from 26.42 (95% CI: 26.16–26.68) in 1999 to 11.12 (95% CI: 10.98–11.27) in 2025, with an AAPC of −3.38 (95% CI: −4.13 to −2.63, *p* < 0.001); however, White adults experienced a significant increase from 2018 to 2021 before mortality declined again through 2025. Overall, although mortality improved across all racial and ethnic groups, persistent disparities remained, particularly among Black adults, who consistently had the highest mortality burden (Figure 3, Table 1, Supplementary Tables S3, S4).

### 3.4 Urbanization

Urbanization-stratified analyses, available for 1999–2020, showed higher mortality in non-metropolitan than metropolitan areas. Non-metropolitan areas had an average AAMR of 18.54, compared with 15.27 in metropolitan areas. In metropolitan areas, AAMR decreased from 26.22 (95% CI: 25.96–26.49) in 1999 to 11.02 (95% CI: 10.88–11.16) in 2020, with an AAPC of −4.29 (95% CI: −4.98 to −3.61, *p* < 0.001). Mortality declined significantly from 1999 to 2009 and again from 2009 to 2018, before increasing significantly from 2018 to 2020 (APC: 7.31; 95% CI: 0.004–15.16, *p* = 0.050). As with the overall 2018–2021 inflection, the metropolitan 2018–2020 increase rests on a borderline *p*-value with a confidence interval lower bound near zero and should be interpreted as a provisional signal.

**Figure 4:**
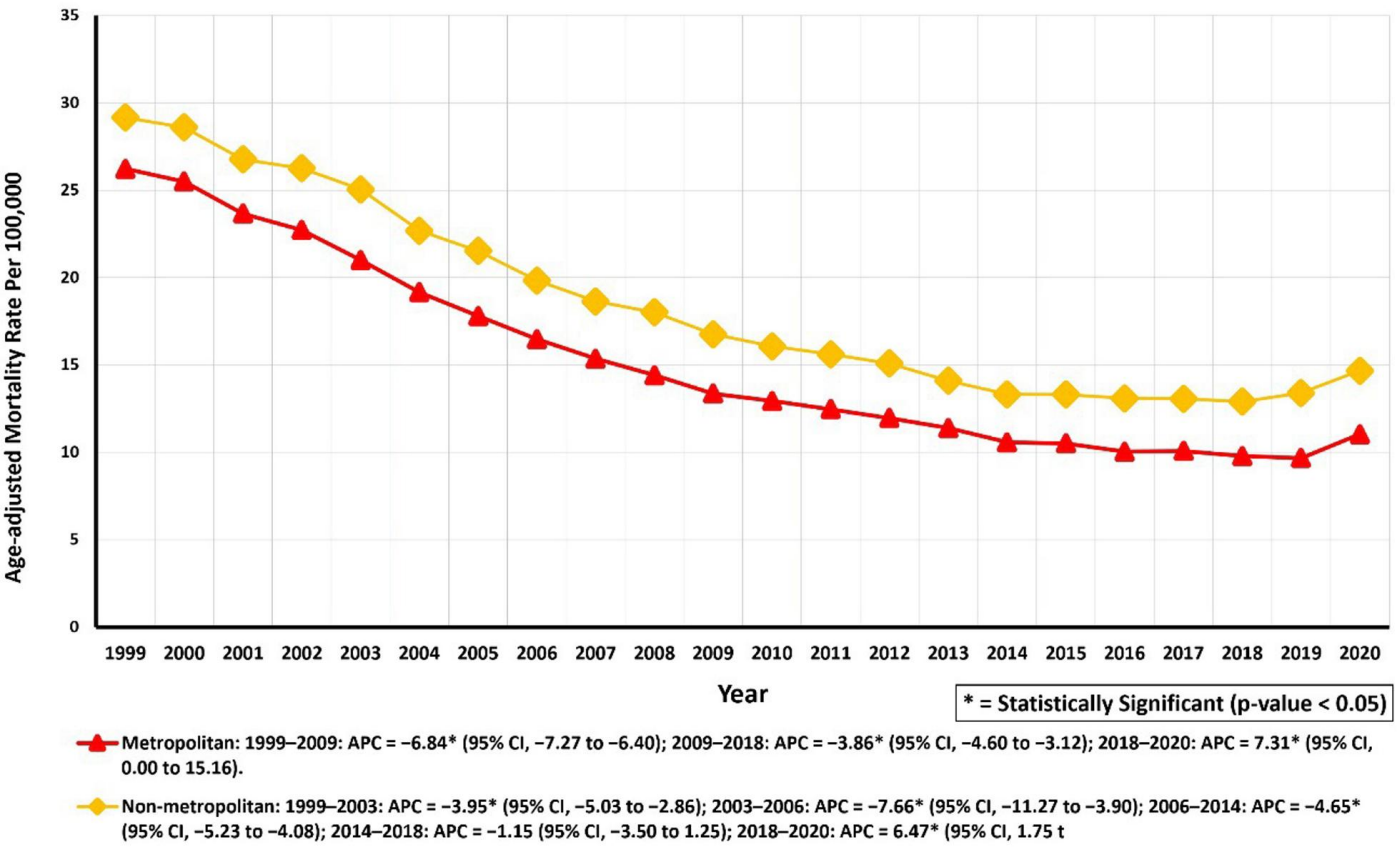
Urbanization-stratified age-adjusted mortality rates per 100,000 for mortality involving coexisting ASCVD- and stroke-related conditions among U.S. adults aged ≥25 years, 1999–2020.

In non-metropolitan areas, AAMR decreased from 29.16 (95% CI: 28.59–29.74) in 1999 to 14.66 (95% CI: 14.30–15.02) in 2020, corresponding to an AAPC of −3.29 (95% CI: −4.03 to −2.54, *p* < 0.001). Declines were significant from 1999 to 2003, 2003–2006, and 2006–2014, followed by a stable period from 2014 to 2018 and a significant increase from 2018 to 2020 (APC: 6.47; 95% CI: 1.75–11.41, *p* = 0.013). These findings indicate that rural populations maintained a higher mortality burden throughout the study period, despite significant long-term improvements in both metropolitan and non-metropolitan settings (Figure 4, Table 1, Supplementary Table S5).

### 3.5 Census region

**Figure 5:**
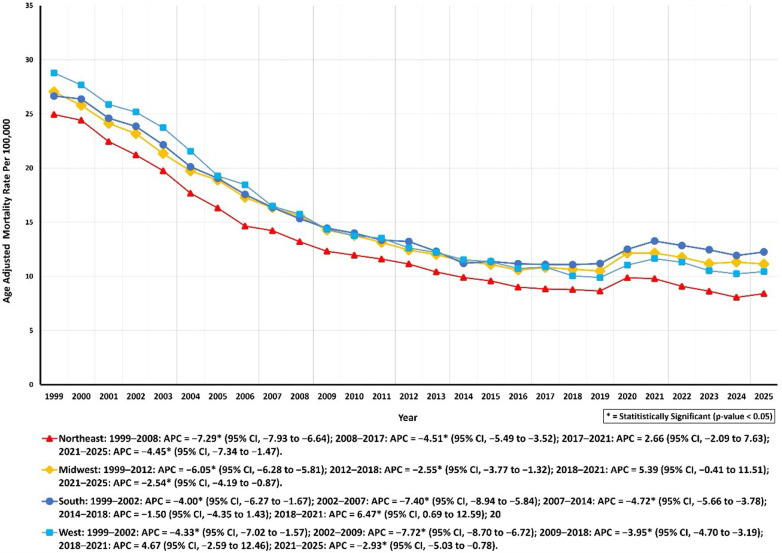
Census region-stratified age-adjusted mortality rates per 100,000 for mortality involving coexisting ASCVD- and stroke-related conditions among U.S. adults aged ≥25 years, 1999–2025.

Regional analyses demonstrated persistent geographic variation in mortality burden. The South had the highest average AAMR (15.62), followed by the West (15.50) and Midwest (15.16), while the Northeast had the lowest average AAMR (13.13). In the Northeast, AAMR declined from 24.94 (95% CI: 24.43–25.44) in 1999 to 8.40 (95% CI: 8.15–8.66) in 2025, corresponding to an AAPC of −4.41 (95% CI: −5.27 to −3.55, *p* < 0.001). In the Midwest, AAMR decreased from 27.03 (95% CI: 26.54–27.53) to 11.12 (95% CI: 10.85–11.40), with an AAPC of −3.45 (95% CI: −4.13 to −2.76, *p* < 0.001). In the South, AAMR declined from 26.65 (95% CI: 26.24–27.06) to 12.25 (95% CI: 12.04–12.46), with an AAPC of −3.07 (95% CI: −3.89 to −2.24, *p* < 0.001), although this region experienced a significant increase from 2018 to 2021 (APC: 6.47; 95% CI: 0.69–12.59, *p* = 0.031) before mortality declined again from 2021 to 2025. In the West, AAMR fell from 28.78 (95% CI: 28.21–29.35) in 1999 to 10.43 (95% CI: 10.18–10.69) in 2025, with an AAPC of −3.92 (95% CI: −4.81 to −3.02, *p* < 0.001). Thus, all regions experienced significant long-term declines, but the Northeast achieved the greatest relative improvement, whereas the South retained the highest mortality burden over time (Figure 5, Table 1, Supplementary Table S6).

### 3.6 Place of death

**Figure 6:**
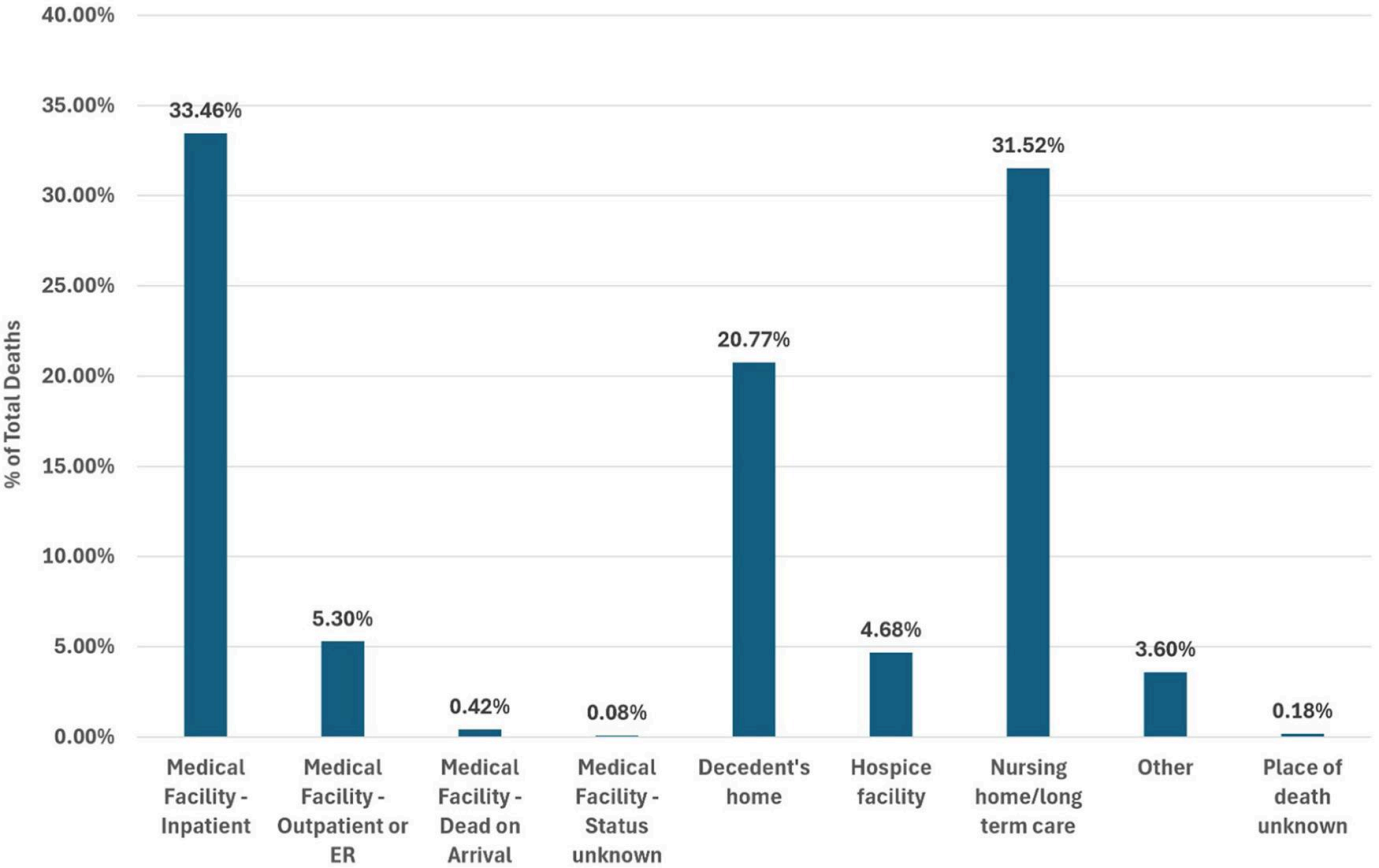
Place-of-death trends in the United States, highlighting mortality involving coexisting ASCVD- and stroke-related conditions among U.S. adults aged ≥25 years, 1999–2025.

Place-of-death analyses showed that the largest proportion of deaths occurred in medical facility inpatient settings (33.46%), followed by nursing home or long-term care facilities (31.52%) and the decedent's home (20.77%). Smaller proportions occurred in medical facility outpatient or emergency room settings (5.30%) and hospice facilities (4.68%), whereas deaths categorized as other locations accounted for 3.60% of all deaths. Only a small fraction occurred as dead-on-arrival (0.42%), at an unknown place of death (0.18%), or in facilities with status unknown (0.08%). Overall, these findings suggest that most deaths involving coexisting ASCVD- and stroke-related conditions occurred in institutional or residential care settings rather than in outpatient or undefined locations (Figure 6, Supplementary Table S7).

### 3.7 State-level variation

**Figure 7:**
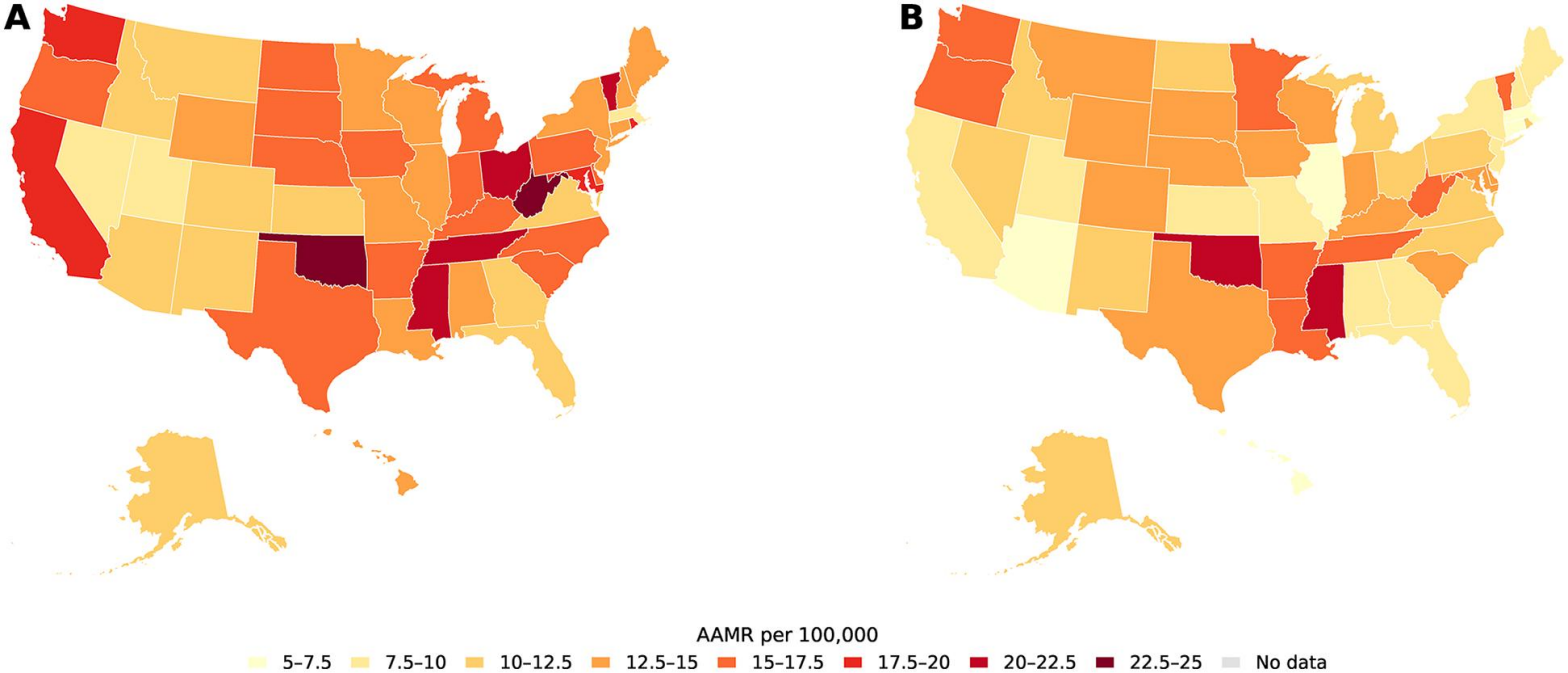
**(A)** state-stratified age-adjusted mortality rates per 100,000 for mortality involving coexisting ASCVD- and stroke-related conditions among U.S. adults aged ≥25 years, 1999–2020. **(B)** State-stratified age-adjusted mortality rates per 100,000 for mortality involving coexisting ASCVD- and stroke-related conditions among U.S. adults aged ≥25 years, 2021–2025.

Considerable heterogeneity was observed across U.S. states. During 1999–2020, West Virginia had the highest average AAMR (24.26), whereas Utah had the lowest (8.97). States in the highest mortality decile included West Virginia, Mississippi, Oklahoma, Vermont and Tennessee, while states in the lowest decile included Utah, Massachusetts, Nevada, Colorado, and Arizona. In the later period, 2021–2025, Mississippi had the highest AAMR (21.62), whereas Connecticut had the lowest (5.67). The highest-burden states during this interval included Mississippi, Oklahoma, Louisiana, and West Virginia, while the lowest-burden states included Connecticut, Massachusetts, Utah, New York, and Illinois. These findings indicate persistent state-level disparities, with a concentration of elevated mortality in parts of the South and Appalachia, and comparatively lower mortality in northeastern and selected western states (Figures 7a,b, Supplementary Table S8).

### 3.8 Sensitivity analyses

Sensitivity analyses based on alternative cause-of-death definitions confirmed the overall stability of the primary findings (Supplementary Table S9, Panel A). For UCD ASCVD, mortality declined significantly overall, with an AAPC of −2.75 (95% CI: −3.15 to −2.36, *p* < 0.001). The steepest reduction occurred from 2003 to 2010 (APC: −4.58; 95% CI: −5.06 to −4.10), followed by continued decline from 2010 to 2018 (APC: −2.29; 95% CI: −2.70 to −1.88), a non-significant increase during 2018–2021 (APC: 1.55; 95% CI: −1.50 to 4.69, *p* = 0.295), and a significant decline from 2021 to 2025 (APC: −3.34; 95% CI: −4.27 to −2.41). For UCD Stroke, the overall trend also showed a significant decline (AAPC: −2.90; 95% CI: −3.42 to −2.37, *p* < 0.001), with the sharpest decrease between 2002 and 2006 (APC: −6.45; 95% CI: −7.98 to −4.89). The 2018–2021 increase was non-significant for this definition (APC: 0.96; 95% CI: −2.65 to 4.71, *p* = 0.571). For UCD ASCVD with Stroke, a stronger overall decline was observed (AAPC: −4.58; 95% CI: −5.15 to −4.01, *p* < 0.001), although the rate of improvement attenuated markedly after 2016 (APC: −0.94; 95% CI: −1.71 to −0.17); no joinpoint was identified in the 2018–2021 window for this definition. For UCD Stroke with ASCVD, mortality declined significantly overall (AAPC: −3.13; 95% CI: −4.20 to −2.05, *p* < 0.001), despite a non-significant increase from 2018 to 2021 (APC: 5.59; 95% CI: −3.65 to 15.70, *p* = 0.226) and a non-significant decline thereafter (APC: −1.73; 95% CI: −4.31 to 0.93, *p* = 0.185). Across all four alternative definitions, the 2018–2021 inflection was either non-significant or absent as a joinpoint segment, consistent with the borderline nature of the primary finding.

Restriction of the primary ASCVD definition to atherosclerotic-specific codes (I25.x) co-listed with stroke codes (I60, I61, I63, I64, I69.0, I69.1, I69.3, and I69.4) identified 834,046 deaths over 1999–2025, approximately 4.8% fewer than the primary analysis (Supplementary Table S9, Panel B). The overall AAMR declined from 26.24 (95% CI: 26.00–26.48) in 1999 to 10.06 (95% CI: 9.95–10.18) in 2025, with an AAPC of −3.75 (95% CI: −4.47 to −3.03, *p* < 0.001). The 2018–2021 increase under this restricted definition was non-significant in the overall population (APC: +5.74; 95% CI: −0.08 to 11.90, *p* = 0.053), confirming that the borderline inflection in the primary analysis is not explained by inclusion of the non-specific circulatory codes.

Sex-stratified analyses under the I25.x-restricted definition were consistent with these overall findings. Among females (414,982 deaths), AAMRs declined significantly across four segments from 1999 to 2018, followed by a non-significant increase during 2018–2021 (APC: +5.10; 95% CI: −0.90 to 11.46, *p* = 0.090) and a significant decline from 2021 to 2025 (APC: −3.84; 95% CI: −5.64 to −2.00, *p* < 0.001), yielding an AAPC of −4.44 (95% CI: −5.16 to −3.72, *p* < 0.001). Among males (419,064 deaths), mortality declined across two segments from 1999 to 2018 — a steeper early reduction from 1999 to 2010 (APC: −6.18; 95% CI: −6.61 to −5.75) followed by a shallower decline from 2010 to 2018 (APC: −2.70; 95% CI: −3.67 to −1.71) — then showed a non-significant increase during 2018–2021 (APC: +5.94; 95% CI: −1.43 to 13.87, *p* = 0.109) and a significant decline from 2021 to 2025 (APC: −3.24; 95% CI: −5.30 to −1.15, *p* = 0.005), with an overall AAPC of −3.32 (95% CI: −4.18 to −2.45, *p* < 0.001). Females showed a steeper long-term decline than males under both the primary and I25.x-restricted definitions.

Replication of the primary analysis using confirmed 1999–2024 data only, excluding provisional 2025 observations, yielded 845,703 deaths and a stronger overall 2018–2021 signal (APC: +6.50; 95% CI: 1.01 to 12.30, *p* = 0.024), with an AAPC of −3.66 (95% CI: −4.36 to −2.95, *p* < 0.001) (Supplementary Table S9, Panel B). However, sex-stratified estimates for this confirmed-data series remained non-significant: females showed an APC of +5.90 (95% CI: −0.26 to 12.45, *p* = 0.059) and an AAPC of −4.27 (95% CI: −5.04 to −3.50, *p* < 0.001) over 424,303 deaths, while males showed an APC of +6.71 (95% CI: −0.17 to 14.05, *p* = 0.055) and an AAPC of −3.27 (95% CI: −4.12 to −2.42, *p* < 0.001) over 421,400 deaths. The strengthening of the overall 2018–2021 signal following exclusion of provisional 2025 data, combined with the persistence of non-significant sex-stratified estimates, further supports a borderline and sex-heterogeneous inflection rather than a sustained acceleration in vascular mortality (Table 1, Supplementary Table S9).

## 4 Discussion

This retrospective study analyzed 876,383 deaths involving coexisting ASCVD- and stroke-related conditions among adults aged ≥25 years using the CDC WONDER Multiple Cause of Death database. Age-adjusted mortality rates (AAMRs) declined from 26.82 per 100,000 in 1999 to 10.91 per 100,000 in 2025 (AAPC −3.53%; *p* < 0.001). Declines occurred through 2002 (APC −4.87%), accelerated from 2002 to 2009 (APC −7.16%), and continued from 2009 to 2018 (APC −3.54%), but a borderline significant increase occurred during 2018–2021 (APC +6.06%; 95% CI, 0.01–12.48; *p* = 0.050), which was not replicated under stricter cause-of-death definitions and should be interpreted as a modest inflection rather than a robust acceleration, followed by another decline through 2025. Males had higher mortality than females, older adults carried the greatest burden, younger adults experienced an unfavourable increase after 2015, and mortality remained concentrated in the South, West, non-metropolitan areas, and among Black or African American populations. Most deaths occurred in inpatient or long-term care settings, and state-level patterns showed persistent hotspots in Appalachia and the Deep South. Sensitivity analyses using alternative cause-of-death definitions showed similar broad temporal patterns, though the 2018–2021 increase was non-significant across all four alternative definitions, consistent with a borderline rather than robust inflection in the primary analysis.

Viewed through a cardiovascular epidemiology and vascular medicine lens, the principal finding is that mortality involving coexisting ASCVD- and stroke-related conditions in the United States declined substantially over more than two decades, but this progress slowed and became vulnerable to reversal in the late 2010s. The overall AAMR decreased by approximately 59% from 1999 to 2025, with the steepest reductions occurring before 2018, a borderline significant increase during 2018–2021 that attenuated under stricter cause-of-death definitions, and renewed decline thereafter. This pattern is consistent with broader national data showing long-term improvement in cardiovascular mortality followed by attenuation of gains and recent adverse inflections. An analysis of atherosclerotic heart disease deaths from 1999 to 2020 showed a marked decline in AAMR, but with abrupt increases between 2018 and 2020 (1). Similarly, a 50-year assessment of premature heart disease mortality found that progress has stagnated since 2011, particularly among men and Black adults (2). Updated national data also demonstrated a sharp rise in cardiovascular disease mortality during the COVID-19 era, contributing to substantial excess deaths (28). These contextual findings support the interpretation that the borderline reversal observed in our study reflects a broader slowing of progress in cardiovascular prevention rather than an isolated anomaly, though the modest and code-sensitive nature of the 2018–2021 signal in the present analysis warrants caution in attributing it to specific pandemic-era mechanisms.

Several mechanisms may underlie this unfavorable late-period inflection. Earlier declines in vascular mortality likely reflected improvements in smoking reduction, lipid lowering, blood pressure control, acute reperfusion strategies, and secondary prevention. In contrast, the more recent slowdown likely reflects a combination of worsening obesity, diabetes, and hypertension burden; stagnation in ASCVD risk-factor treatment and control; persistent gaps in preventive care; structural and socioeconomic inequities; and disruptions in health-care access during the COVID-19 pandemic (29, 30). The COVID-era causal attribution should be interpreted cautiously in this study, however, because the 2018–2021 increase in the primary analysis rested on a borderline *p*-value and a confidence interval lower bound near zero, and was absent or non-significant under all four alternative cause-of-death definitions. The pandemic likely contributed to the observed pattern, supported by national evidence of excess cardiovascular mortality across multiple COVID-19 waves, but its specific contribution cannot be quantified from death-certificate data alone (30). In this context, our findings suggest that prior advances in cardiovascular prevention have not been uniformly sustained across the U.S. population.

Within this study, stroke should be interpreted as part of the operational death-certificate phenotype rather than as a separately adjudicated neurologic endpoint. The observed mortality pattern involving stroke-related coding nonetheless corroborates the broader vascular mortality signal: long-term decline, attenuation of progress, and a later adverse inflection. Prior national analyses of stroke mortality among young adults showed that rates declined until approximately 2010 and then increased again from 2013 to 2019 (4). A population-based study of atrial fibrillation-related stroke mortality likewise found reductions through 2008 followed by subsequent increases, including sharp rises in 2019–2020 (31). More recent analyses extending through 2024 reported declines until 2018, a spike during 2018–2021 and decline thereafter (32). Similarly, a five-decade analysis of ischemic stroke mortality demonstrated substantial long-term decline but reported steep increases after 2014 (33). Thus, although the present analysis cannot establish clinically adjudicated concurrent ASCVD and stroke in individual decedents, the inclusion of stroke-related coding strengthens the interpretation that the observed death-certificate phenotype reflects a broader adverse vascular-risk environment.

The age-stratified findings further support this framework. Mortality remained highest among adults aged ≥65 years, whose AAMR fell from 128.1 in 1999 to 48.27 in 2025 (AAPC −3.83%). Adults aged 45–64 years experienced only modest long-term decline and showed a sharp increase between 2017 and 2021, while the 25–44-year group demonstrated a significant increase in AAMR after 2015 (APC +4.45%). These findings should be interpreted with caution given the small absolute death counts in the 25–44-year group and the low absolute AAMR values throughout the study period; the observed increase is statistically significant but reflects a directional signal warranting surveillance rather than a stable quantitative estimate. The young-adult and middle-aged signal has been observed in prior stroke-focused studies, which reported rising mortality among adults aged 25–34 and 35–44 years after earlier improvements (4), levelling or worsening trends among adults aged 35–64 years at the county level (34), increasing relevance of modifiable vascular risk factors such as smoking, hypertension, and dyslipidemia among younger adults (35), and less favourable stroke-incidence trends in younger compared with older adults across high-income countries (36). Other recent mortality analyses involving stroke and diabetes or atrial fibrillation have reported similar age-specific reversals, particularly around 2018–2021 (31, 32). Taken together, these observations suggest that the recent resurgence in mortality among younger and middle-aged adults reflects worsening vascular-risk exposure and incomplete prevention across the life course.

Shared demographic and geographic disparities were a central feature of our findings. Mortality was consistently higher among men than women, among Black individuals compared with other racial and ethnic groups, among residents of the South and West, and among those living in non-metropolitan areas. These disparities mirror broader national patterns in cardiovascular and stroke mortality. Men have persistently higher cardiovascular mortality rates than women in analyses of atherosclerotic heart disease and premature heart disease death (1, 2), while more recent studies indicate that both sexes experienced adverse changes during the late 2010s and early 2020s (4, 31, 32). Racial disparities were also pronounced. Black individuals had the highest average AAMR in our cohort, and previous studies have similarly documented persistently higher cardiovascular mortality among Black adults compared with White adults, as well as the highest stroke-related mortality among non-Hispanic Black adults, with more recent reversals or plateauing in multiple racial and ethnic groups (4, 31, 32, 37). The wide confidence intervals around American Indian and Alaska Native AAMR estimates in this study reflect smaller population denominators rather than data instability, as annual death counts in this group exceeded 100 throughout the study period; nonetheless, these estimates should be interpreted cautiously given the documented misclassification of race on death certificates in this population (17–19). Geographic disparities were likewise substantial. The South had the highest regional burden in our study, followed closely by the West and Midwest, while the Northeast consistently had the lowest rates. These findings are consistent with prior evidence identifying the South, the rural Stroke Belt, and parts of Appalachia as persistent high-burden areas for vascular mortality (4, 32, 34). The higher mortality observed in non-metropolitan populations also aligns with national reports showing that rural communities carry higher mortality and slower improvements for ischemic heart disease, heart failure, and stroke (7, 38). Collectively, these patterns suggest that the recent slowing of progress in vascular mortality has occurred on top of longstanding inequities in risk-factor burden, health-care access, structural disadvantage, structural racism, and preventive care delivery (39).

The place-of-death and state-level analyses add further context to these disparities. Most deaths occurred in inpatient facilities or long-term care settings, with a substantial minority occurring at home. Prior stroke-related mortality studies have also shown that most deaths occur in medical facilities, but that home deaths have increased over time and may reflect disparities in access to specialized care and end-of-life services (4, 32, 40). At the state level, the concentration of high AAMRs in West Virginia, Mississippi, Oklahoma, Louisiana, and neighboring states reinforces the persistence of vascular mortality hotspots in Appalachia and the Deep South, whereas lower rates in Connecticut, Massachusetts, Utah, and other northeastern or mountain states suggest more favorable regional prevention and care environments. These state-level patterns are broadly consistent with prior national mortality reports and emphasize that geographic inequities remain deeply embedded in the U.S. vascular mortality landscape (4, 31).

The sensitivity analyses further strengthen the overall interpretation. When deaths with ASCVD as the underlying cause were examined separately, mortality still declined significantly, showed its steepest reduction during 2003–2010, and demonstrated a non-significant late increase followed by renewed decline. A similar pattern was observed when stroke was analyzed as the underlying cause. For deaths in which ASCVD and stroke were both listed, the decline was steeper overall but slowed notably after 2016. Critically, the 2018–2021 increase was non-significant across all four alternative definitions (UCD ASCVD *p* = 0.29; UCD Stroke *p* = 0.57; UCD Stroke-with-ASCVD *p* = 0.23; absent as a joinpoint segment for UCD ASCVD-with-Stroke), indicating that this inflection does not replicate under stricter cause-of-death classifications. Additionally, restriction of the primary ASCVD definition to atherosclerotic-specific codes (I25.x) identified 834,046 deaths, approximately 4.8% fewer than the primary analysis and produced a near-identical 4-joinpoint structure with an AAPC of −3.75% (95% CI: −4.47 to −3.03), compared with −3.53% in the primary analysis. Critically, under this restricted definition the 2018–2021 segment was non-significant across all three strata (overall *p* = 0.053; females *p* = 0.090; males *p* = 0.109), confirming that the non-specific circulatory codes do not explain the primary findings and that the borderline 2018–2021 inflection does not strengthen under a stricter atherosclerotic-specific definition. Exclusion of provisional 2025 data yielded a stronger 2018–2021 overall APC (+6.50%; *p* = 0.024) but left sex-stratified estimates non-significant, further supporting a modest and sex-heterogeneous inflection. These findings indicate that alternative operational definitions based on death-certificate coding did not materially alter the central temporal pattern of the analysis, while appropriately calibrating the strength of the 2018–2021 signal.

Recent comorbidity-focused cardiovascular mortality studies further support these findings. Mortality related to peripheral artery disease with hyperlipidemia increased substantially and showed marked sex, racial, and regional disparities (41). Deaths involving atrial fibrillation/flutter with heart failure also rose over time, with important differences across sex, race/ethnicity, and region (42), while national arrhythmia- and heart failure-related mortality analyses demonstrated a growing burden, particularly among men, American Indian/Alaska Native populations, and rural communities (43). Heart failure mortality associated with tobacco use disorder increased sharply and disproportionately affected American Indian/Alaska Native adults and rural states (44). In contrast, chronic ischemic heart disease mortality among adult cancer patients declined overall but remained higher in older adults, men, and non-Hispanic Black individuals (45). Cardiovascular mortality among individuals with pulmonary fibrosis more than doubled, with steeper increases among males and White adults (46). Mortality involving atrial fibrillation and rheumatic heart disease also increased, particularly among women and non-Hispanic White adults, with most deaths occurring in inpatient settings (47). Studies of ischemic heart disease disparities similarly documented persistently higher mortality in rural populations, older adults, and selected racial groups (48). Atrial fibrillation-related mortality among older adults with obstructive sleep apnea rose rapidly, especially among those aged ≥85 years, women, rural residents, and White individuals (49). Sepsis-associated cardiovascular mortality declined initially but increased sharply after 2019, with the greatest burden among men, non-Hispanic Black adults, older individuals, and residents of the South and West (50). Although these conditions differ clinically, the recurring epidemiologic pattern is consistent: slowing gains in mortality reduction, persistent demographic and geographic disparities, and evidence that progress in one cardiovascular domain may be offset by worsening trends in another. These studies also share the same methodological framework and database, which strengthens cross-study comparability but also means that the reference cluster reflects a common set of analytical choices rather than independent replication across diverse methodologies. Broader contextual sources, including primary risk-factor epidemiology and AHA statistical updates, are incorporated where available to provide a more balanced literature base (9, 29).

Overall, the findings of this study have important implications for prevention and policy. The borderline interruption of long-term mortality declines during 2018–2021, the emerging signal among younger adults, and the persistence of racial, regional, and rural disparities all indicate that progress in reducing vascular mortality has been substantial but uneven and vulnerable to reversal. These findings are particularly concerning in light of projected increases in cardiovascular risk-factor burden and future cardiovascular and stroke prevalence in the United States through 2050 (51). Strengthening primary and secondary prevention will require renewed attention to traditional cardiovascular risk-factor control, earlier identification of high-risk individuals, and more equitable delivery of preventive, acute, and longitudinal care. Public health strategies should prioritize high-burden states and regions, expand access to preventive and acute cardiovascular care in rural communities, improve risk-factor management among younger and middle-aged adults, and address structural inequities that continue to shape vascular outcomes across the United States.

## 5 Limitations

This study has several limitations. First, it was based on the CDC WONDER Multiple Cause of Death database and therefore depended on the accuracy of death certificate completion and ICD-10 coding; miscoding or misclassification of both underlying and contributing causes of death may have affected case ascertainment and temporal trends. Second, as a mortality-based analysis, this study could not distinguish whether observed changes were driven by differences in disease incidence, case-fatality, or both. Third, the database lacked detailed clinical and treatment-related covariates, including cardiovascular risk factors, comorbidities, disease severity, medication use, procedures, healthcare access, and quality of care, which limited adjustment for potential confounding and prevented mechanistic interpretation of the observed disparities. Fourth, although the ICD-10 code list followed prior CDC WONDER studies, inclusion of several non-specific circulatory codes (e.g., I51.7, I51.9, I87.8, I87.9, I99, and R93.1) could introduce minor misclassification or overestimation of atherosclerotic mortality. To address this, ICD-10 codes were cross-checked against prior large-scale epidemiological studies using identical code sets, including Parvez et al. (13), and an internal sensitivity analysis restricted to ischemic heart disease-specific codes (I25.x) was conducted to ensure consistency. Trends remained stable under this restricted definition, supporting the robustness of the primary findings. Fifth, race and ethnicity recorded on death certificates may be misclassified, particularly among American Indian and Alaska Native populations, and these findings should therefore be interpreted with caution. Sixth, secular changes in diagnostic awareness, coding practices, and completeness of death certificate reporting over time may have influenced the observed trends, particularly in a multiple-cause-of-death analysis. Seventh, the primary outcome represented mortality involving coexisting ASCVD- and stroke-related conditions as documented on death certificates, rather than clinically adjudicated concurrent disease; accordingly, the findings should be interpreted as reflecting a death-certificate-defined mortality phenotype rather than confirmed simultaneous vascular diagnoses in individual decedents. Eighth, special caution is warranted for the most recent years because provisional 2025 mortality data were included; these data remain subject to revision and possible reporting delays, and the most recent trend estimates should therefore be interpreted as preliminary. A sensitivity analysis excluding provisional 2025 data confirmed that the overall trend structure was preserved, though sex-stratified 2018–2021 estimates remained non-significant. Ninth, the geographic analyses were ecological and cannot establish causality at the individual level. Finally, because urbanization analyses were limited to the archived 1999–2020 file for temporal consistency, urban-rural patterns were not extended into the later period; these findings should therefore be interpreted as reflecting the long-term pre-2021 trend rather than the full 1999–2025 study interval. Despite these limitations, the large national scope and consistency of the findings across subgroup and alternative definition analyses support the overall robustness of the observed mortality patterns.

## 6 Conclusion

Although mortality involving coexisting ASCVD- and stroke-related conditions in the United States declined markedly from 1999 to 2025, these gains slowed over time, were interrupted by a borderline reversal during 2018–2021 that attenuated under stricter cause-of-death definitions, and remained unevenly distributed across demographic and geographic groups. The stroke-related component of this death-certificate-defined mortality phenotype tracked closely with the broader vascular mortality signal, consistent with shared vascular risk at the population level. The persistent burden among men, older adults, Black individuals, southern and non-metropolitan populations, and the recent increase among younger adults highlight the need for stronger and more equitable prevention strategies. Renewed efforts to improve cardiovascular risk-factor control, expand access to high-quality care, and target high-burden populations and regions will be critical to prevent further erosion of progress in vascular mortality.

## Data Availability

The datasets presented in this study can be found in online repositories. The names of the repository/repositories and accession number(s) can be found in the article/Supplementary Material.

## References

[1] Akhtar M Ashraf DA Nadeem MS Maryam A Ahmed H Akhtar M. Trends in atherosclerotic heart disease-related mortality among U.S. adults aged 35 and older: a 22-year analysis. Int J Cardiol Cardiovasc Risk Prev. (2025) 24:200374. 10.1016/j.ijcrp.2025.200374 40026602 PMC11872109

[2] Ritchey MD Wall HK George MG Wright JS. US Trends in premature heart disease mortality over the past 50 years: where do we go from here? Trends Cardiovasc Med. (2020) 30(6):364–74. 10.1016/j.tcm.2019.09.005 31607635 PMC7098848

[3] Woodruff RC Tong X Khan SS Shah NS Jackson SL Loustalot F. Trends in cardiovascular disease mortality rates and excess deaths, 2010-2022. Am J Prev Med. (2024) 66(4):582–9. 10.1016/j.amepre.2023.11.009 37972797 PMC10957309

[4] Ariss RW Minhas AMK Lang J Ramanathan PK Khan SU Kassi M. Demographic and regional trends in stroke-related mortality in young adults in the United States, 1999 to 2019. J Am Heart Assoc. (2022) 11(18):e025903. 10.1161/JAHA.122.025903 36073626 PMC9683653

[5] Yang Q Tong X Schieb L Vaughan A Gillespie C Wiltz JL. Vital signs: recent trends in stroke death rates—united States, 2000-2015. MMWR Morb Mortal Wkly Rep. (2017) 66(35):933–9. 10.15585/mmwr.mm6635e1 28880858 PMC5689041

[6] Yahya T Jilani MH Khan SU Mszar R Hassan SZ Blaha MJ. Stroke in young adults: current trends, opportunities for prevention and pathways forward. Am J Prev Cardiol. (2020) 3:100085. 10.1016/j.ajpc.2020.100085 34327465 PMC8315351

[7] Khan MS Kumar P Sreenivasan J Khan SU Nasir K Mehra MR. Rural-urban differences in mortality from ischemic heart disease, heart failure, and stroke in the United States. Circ Cardiovasc Qual Outcomes. (2021) 14(4):e007341. 10.1161/CIRCOUTCOMES.120.007341 33877879 PMC8059762

[8] Kim W Kim EJ. Heart failure as a risk factor for stroke. J Stroke. (2018) 20(1):33–45. 10.5853/jos.2017.02810 29402070 PMC5836579

[9] Martin SS Aday AW Almarzooq ZI Anderson CAM Arora P Avery CL. Heart disease and stroke statistics-2024 update: a report of US and global data from the American Heart Association. Circulation. (2024) 149(8):e347–913. 10.1161/CIR.0000000000001209 38264914 PMC12146881

[10] Friede A Reid JA Ory HW. CDC WONDER: a comprehensive on-line public health information system of the centers for disease control and prevention. Am J Public Health. (1993) 83(9):1289–94. 10.2105/AJPH.83.9.1289 8395776 PMC1694976

[11] Centers for Disease Control and Prevention. CDC WONDER: Wide-ranging Online Data for Epidemiologic Research. Available online at: https://wonder.cdc.gov/ (Accessed March 29, 2026).

[12] World Health Organization. International Statistical Classification of Diseases and Related Health Problems, 10th Revision (ICD-10). Geneva: World Health Organization (2004).

[13] Parvez A Zeeshan N Sultan L Mukhlis M Bhagwan R Munir T. Trends and disparities in obesity and atherosclerotic cardiovascular disease mortality in a 25-year retrospective analysis. Sci Rep. (2026) 16(1):844. 10.1038/s41598-025-30451-1 PMC12780052 41453910

[14] Bilal AR Sajid M Qureshi S Gaba H Ahmad R Alta’amreh M. Temporal trends in heart failure and stroke-related mortality in the United States, 1999-2023. J Stroke Cerebrovasc Dis. (2026) 35(3):108538. 10.1016/j.jstrokecerebrovasdis.2025.108538 41475590

[15] U.S. Census Bureau. Census regions and divisions of the United States [Internet]. Washington (DC): U.S. Census Bureau. Available online at: https://www.census.gov/geographies/reference-maps/2010/geo/2010-census-regions-and-divisions-of-the-united-states.html (Accessed March 2, 2026).

[16] Ingram DD Franco SJ. 2013 NCHS urban-rural classification scheme for counties. Vital Health Stat 2. (2014) (166):1–73. https://www.cdc.gov/nchs/data/series/sr_02/sr02_166.pdf. 24776070

[17] Arias E Heron M Hakes JK. The validity of race and hispanic-origin reporting on death certificates in the United States: an update. Vital Health Stat 2. (2016) (172):1–21. 28436642

[18] Arias E Schauman WS Eschbach K Sorlie PD Backlund E. The validity of race and hispanic origin reporting on death certificates in the United States. Vital Health Stat 2. (2008) (148):1–23. 19024798

[19] Jim MA Arias E Seneca DS Hoopes MJ Jim CC Johnson NJ. Racial misclassification of American Indians and Alaska natives by Indian health service contract health service delivery area. Am J Public Health. (2014) 104(3):S295–302. 10.2105/AJPH.2014.301933 24754617 PMC4035863

[20] Tiwari C Beyer K Rushton G. The impact of data suppression on local mortality rates: the case of CDC WONDER. Am J Public Health. (2014) 104(8):1386–8. 10.2105/AJPH.2014.301900 24922161 PMC4103252

[21] Quick H. Estimating county-level mortality rates using highly censored data from CDC WONDER. Prev Chronic Dis. (2019) 16:E76. 10.5888/pcd16.180441 31198162 PMC6583819

[22] Anderson RN Rosenberg HM. Age standardization of death rates: implementation of the year 2000 standard. Natl Vital Stat Rep. (1998) 47(3):1–16. https://www.cdc.gov/nchs/data/nvsr/nvsr47/nvs47_03.pdf. 9796247

[23] Kim HJ Fay MP Feuer EJ Midthune DN. Permutation tests for joinpoint regression with applications to cancer rates. Stat Med. (2000) 19(3):335–51. 10.1002/(sici)1097-0258(20000215)19:3<335::aid-sim336>3.0.co;2-z 10649300 10.1002/(sici)1097-0258(20000215)19:3<335::aid-sim336>3.0.co;2-z

[24] Ingram DD Malec DJ Makuc DM Kruszon-Moran D Gindi RM Albert M. National center for health statistics guidelines for analysis of trends. Vital Health Stat 2. (2018) (179):1–71. https://www.cdc.gov/nchs/data/series/sr_02/sr02_179.pdf. 29775435

[25] Irimata KE Bastian BA Clarke TC Curtin SC Rui P. Guidance for selecting model options in the national cancer institute joinpoint regression software. Vital Health Stat 2. (2022) (194):1–22. 10.15620/cdc:118050 36255743

[26] Clegg LX Hankey BF Tiwari R Feuer EJ Edwards BK. Estimating average annual per cent change in trend analysis. Stat Med. (2009) 28(29):3670–82. 10.1002/sim.3733 19856324 PMC2843083

[27] Von Elm E Altman DG Egger M Pocock SJ Gøtzsche PC Vandenbroucke JP. STROBE Initiative. The STROBE statement: guidelines for reporting observational studies. PLoS Med. (2007) 4(10):e296. 10.1371/journal.pmed.0040296 17941714 PMC2020495

[28] Woodruff RC Tong X Loustalot FV Khan SS Shah NS Jackson SL. Cardiovascular disease mortality trends, 2010–2022: an update with final data. Am J Prev Med. (2025) 68(2):391–5. 10.1016/j.amepre.2024.09.014 39321995 PMC11757076

[29] Li J Zhang J Somers VK Covassin N Zhang L Xu H. Trends and disparities in treatment and control of atherosclerotic cardiovascular disease in US adults, 1999 to 2018. J Am Heart Assoc. (2024) 13(9):e032527. 10.1161/JAHA.123.032527 38639366 PMC11179884

[30] Han L Zhao S Li S Gu S Deng X Yang L. Excess cardiovascular mortality across multiple COVID-19 waves in the United States from march 2020 to march 2022. Nat Cardiovasc Res. (2023) 2(3):322–33. 10.1038/s44161-023-00220-2 39195997

[31] Ahmad O Farooqi HA Ahmed I Jamil A Nabi R Ullah I. Temporal trends in mortality related to stroke and atrial fibrillation in the United States: a 21 year retrospective analysis of CDC WONDER database. Clin Cardiol. (2024) 47(12):e70058. 10.1002/clc.70058 39679968 PMC11648035

[32] Majeed I Kumar H Kumar K Bai N Rajesh F Kumar S. Trends in diabetes and stroke related mortality in the United States, 1999–2024: a population based analysis. Ann Med Surg (Lond). (2025) 88(2):1521–31. 10.1097/MS9.0000000000004601 41675806 PMC12889402

[33] Qasim R Muzammil L Qammar B Dar A Sultan L Raza M. Five decade mortality trends in ischemic stroke in the United States: a CDC WONDER analysis. Brain Behav. (2025) 16:e71177. 10.1002/brb3.71177 PMC12755966 41476024

[34] de Havenon A Zhou LW Johnston KC Dangayach NS Ney J Yaghi S. Twenty year disparity trends in United States stroke death rate by age, race/ethnicity, geography, and socioeconomic status. Neurology. (2023) 101(5):e464–74. 10.1212/WNL.0000000000207446 37258298 PMC10401675

[35] Smajlović D. Strokes in young adults: epidemiology and prevention. Vasc Health Risk Manag. (2015) 11:157–64. 10.2147/VHRM.S53203 25750539 PMC4348138

[36] Scott CA Li L Rothwell PM. Diverging temporal trends in stroke incidence in younger vs older people: a systematic review and meta-analysis. JAMA Neurol. (2022) 79(10):1036–48. 10.1001/jamaneurol.2022.1520 35943738 PMC9364236

[37] Kyalwazi AN Loccoh EC Brewer LPC Ofili EO Xu J Song Y. Disparities in cardiovascular mortality between black and white adults in the United States, 1999 to 2019. Circulation. (2022) 146(3):211–28. 10.1161/CIRCULATIONAHA.122.060199 35861764 PMC9310198

[38] Aggarwal R Chiu N Loccoh EC Kazi DS Yeh RW Wadhera RK. Rural urban disparities: diabetes, hypertension, heart disease, and stroke mortality among black and white adults, 1999–2018. J Am Coll Cardiol. (2021) 77(11):1480–81. 10.1016/j.jacc.2021.01.032 33736831 PMC8210746

[39] Churchwell K Elkind MSV Benjamin RM Carson AP Chang EK Lawrence W. American heart association. Call to action: structural racism as a fundamental driver of health disparities: a presidential advisory from the American heart association. Circulation. (2020) 142(24):e454–68. 10.1161/CIR.0000000000000936 33170755

[40] Lim JK Pagnotta J Lee R Lim DH Breton JM Abecassis ZA. Trends and disparities in ischemic stroke mortality and location of death in the United States: a comprehensive analysis from 1999 to 2020. PLoS One. (2025) 20(4):e0319867. 10.1371/journal.pone.0319867 40202955 PMC11981169

[41] Khan S Hassan M Hussain M Dhedhi HAA Fatima W Nawaz J. Trends and disparities in mortality associated with peripheral artery disease and hyperlipidemia, 1999–2024. Sci Rep. (2025) 15:45008. 10.1038/s41598-025-29224-7 41274931 PMC12748544

[42] Rani S Kumar L Ali SME Ashraf S Bhimani S Kumar S. Trends in United States mortality among patients with atrial fibrillation/flutter related heart failure (1999–2024): disparities by gender, race/ethnicity and region. BMC Cardiovasc Disord. (2025) 25(1):558. 10.1186/s12872-025-05036-5 40739190 PMC12312449

[43] Khan SA Assad AA Qadri M Abbasi SUAM Saleem H Saeed M. National trends in arrhythmias and heart failure related mortality in the United States from 1999 to 2023: a CDC WONDER analysis. J Arrhythm. (2025) 41(6):e70222. 10.1002/joa3.70222 41267929 PMC12628278

[44] Mahmood A Latif F Latif S Smith DN Ayalew BD Raja A. Heart failure and tobacco use disorder: mortality trends and disparities in the United States. Ann Med Surg. (2026) 88(2):1639–47. 10.1097/MS9.0000000000004729 PMC12889312 41675815

[45] Qasim SA Khan I Ul Hassan SS Rath S Khan M Rahman SU. Trends and disparities in chronic ischemic heart disease mortality among adult cancer patients: a nationwide CDC WONDER analysis (1999–2020). Cardiooncology. (2025) 11(1):114. 10.1186/s40959-025-00409-3 41420198 PMC12717754

[46] Ponnada RB Dhruva YV Kumar AV Mootz N Peddada VP Lu C-H. Trends and disparities for cardiovascular-related deaths with underlying pulmonary fibrosis: a retrospective 1999–2020 analysis of CDC WONDER data. Health Sci Rep. (2026) 9(3):e71899. 10.1002/hsr2.71899 41767363 PMC12946471

[47] Hemida MF Saghir M Ibrahim AA Goel A Amir Jalal A Patel K. Temporal trends in mortality involving atrial fibrillation and rheumatic heart disease: a 25 year nationwide analysis. Front Cardiovasc Med. (2025) 12:1687555. 10.3389/fcvm.2025.1687555 41458989 PMC12741100

[48] Karnan N Abdirahman AH Kilaru G Kaur AR Majumder K Fadel LASAA. Assessing disparity in mortality rates for ischemic heart disease using CDC WONDER database: a retrospective analysis. Glob Cardiol Sci Pract. (2025) 2025(1):e202514. 10.21542/gcsp.2025.14 40390997 PMC12085919

[49] Hassan IN Ibrahim M Yaqub S Ibrahim M Abdalla H Aljaili G. Trends in atrial fibrillation related mortality among older adults with obstructive sleep apnea in the United States, 1999–2020. Clin Cardiol. (2025) 48(7):e70178. 10.1002/clc.70178 40662442 PMC12261039

[50] Salman M Cicin J Abdul Jabbar AB El-shaer A Tauseef A Asghar N. Trends in sepsis-associated cardiovascular disease mortality in the United States, 1999 to 2022. Front Cardiovasc Med. (2024) 11:1505905. 10.3389/fcvm.2024.1505905 39717445 PMC11663846

[51] Joynt Maddox KE Elkind MSV Aparicio HJ Commodore-Mensah Y De Ferranti SD Dowd WN. American Heart Association. Forecasting the burden of cardiovascular disease and stroke in the United States through 2050: prevalence of risk factors and disease: a presidential advisory from the American Heart Association. Circulation. (2024) 150(4):e65–88. 10.1161/CIR.0000000000001256 38832505

