# Supplementary material for: Trends and disparities in deaths involving atherosclerotic cardiovascular disease and stroke-related conditions among U.S. adults, 1999–2025
Source: Front Cardiovasc Med. 2026 Jul 16;13:1851946. doi: 10.3389/fcvm.2026.1851946 (PMC13422406; doi:10.3389/fcvm.2026.1851946)
Supplement: Supplementary file 1 [file Datasheet1.docx]

**Trends and Disparities in Deaths Involving Cardiovascular disease** **and Stroke-Related Conditions Among U.S. Adults, 1999–2025**

**Supplementary Figure S1. Case ascertainment Flow diagram**

**Supplementary Table S1.** Trends in Age-Adjusted Mortality Rates and Deaths Involving Coexisting ASCVD- and Stroke-Related Conditions Among United States Adults Aged ≥25 Years, Stratified by Overall and Sex, 1999–2025.

**Supplementary Table S2.** Trends in Age-Adjusted Mortality Rates and Deaths Involving Coexisting ASCVD- and Stroke-Related Conditions Among United States Adults Aged ≥25 Years, Stratified by Age Group, 1999–2025.

**Supplementary Table S3.** Trends in Age-Adjusted Mortality Rates and Deaths Involving Coexisting ASCVD- and Stroke-Related Conditions Among United States Adults Aged ≥25 Years, Stratified by Race and Ethnicity, 1999–2025.

**Supplementary Table S4.** Annual Death Counts for Small-Denominator Subgroups: Adults Aged 25–44 Years and NH American Indian or Alaska Native Adults, 1999–2025.

**Supplementary Table S5.** Trends in Age-Adjusted Mortality Rates and Deaths Involving Coexisting ASCVD- and Stroke-Related Conditions Among United States Adults Aged ≥25 Years, Stratified by Urbanization, 1999–2020.

**Supplementary Table S6.** Trends in Age-Adjusted Mortality Rates and Deaths Involving Coexisting ASCVD- and Stroke-Related Conditions Among United States Adults Aged ≥25 Years, Stratified by Census Region, 1999–2025.

**Supplementary Table S7.** Percentage Distribution of Place of Death Among United States Adults Aged ≥25 Years With Mortality Involving Coexisting ASCVD- and Stroke-Related Conditions, 1999–2025.

**Supplementary Table S8.** Trends in Age-Adjusted Mortality Rates and Deaths Involving Coexisting ASCVD- and Stroke-Related Conditions Among United States Adults Aged ≥25 Years, Stratified by State, 1999–2025.

**Supplementary Table S9.** Sensitivity Analyses of Mortality Involving Coexisting ASCVD- and Stroke-Related Conditions Using Alternative Death-Certificate Definitions.

**Supplementary Figure S1. Case ascertainment Flow diagram**


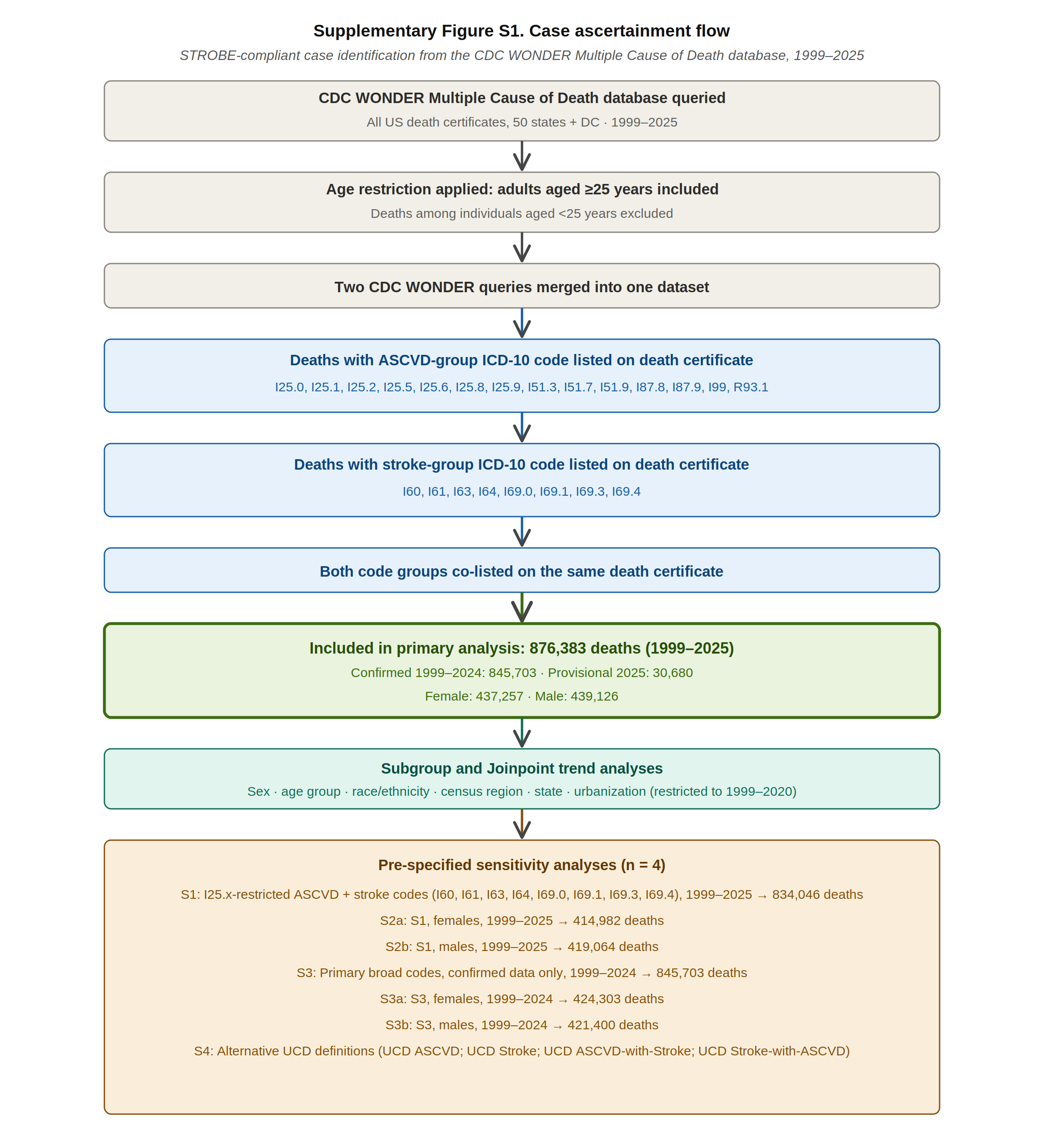


**Supplementary Table S1.** Trends in Age-Adjusted Mortality Rates and Deaths Involving Coexisting ASCVD- and Stroke-Related Conditions Among United States Adults Aged ≥25 Years, Stratified by Overall and Sex, 1999–2025.

| **Year** | **Overall** | | **Women** | | **Men** | |
| --- | --- | --- | --- | --- | --- | --- |
|  | **Deaths** | **AAMR (95% CI)** | **Deaths** | **AAMR (95% CI)** | **Deaths** | **AAMR (95% CI)** |
| **1999** | 47080 | 26.82 (26.57 to 27.06) | 26584 | 23.64 (23.35 to 23.93) | 20496 | 31.29 (30.85 to 31.73) |
| **2000** | 46429 | 26.09 (25.86 to 26.33) | 26089 | 22.86 (22.58 to 23.14) | 20340 | 30.64 (30.22 to 31.07) |
| **2001** | 43925 | 24.29 (24.07 to 24.52) | 24306 | 21.11 (20.84 to 21.37) | 19619 | 28.87 (28.46 to 29.28) |
| **2002** | 42901 | 23.40 (23.17 to 23.62) | 23812 | 20.46 (20.19 to 20.72) | 19089 | 27.60 (27.20 to 28.00) |
| **2003** | 40594 | 21.79 (21.57 to 22.00) | 22266 | 18.91 (18.66 to 19.16) | 18328 | 25.84 (25.46 to 26.22) |
| **2004** | 37475 | 19.82 (19.62 to 20.02) | 20228 | 17.02 (16.78 to 17.25) | 17247 | 23.83 (23.47 to 24.20) |
| **2005** | 35674 | 18.48 (18.29 to 18.68) | 19048 | 15.79 (15.56 to 16.01) | 16626 | 22.34 (22.00 to 22.69) |
| **2006** | 33681 | 17.11 (16.92 to 17.29) | 17836 | 14.54 (14.33 to 14.76) | 15845 | 20.70 (20.37 to 21.03) |
| **2007** | 32089 | 15.96 (15.78 to 16.13) | 17024 | 13.60 (13.40 to 13.81) | 15065 | 19.21 (18.90 to 19.52) |
| **2008** | 30917 | 15.09 (14.92 to 15.26) | 16172 | 12.76 (12.56 to 12.96) | 14745 | 18.36 (18.06 to 18.66) |
| **2009** | 29300 | 13.97 (13.81 to 14.13) | 14938 | 11.55 (11.36 to 11.74) | 14362 | 17.37 (17.08 to 17.65) |
| **2010** | 28745 | 13.49 (13.33 to 13.65) | 14591 | 11.13 (10.95 to 11.31) | 14154 | 16.75 (16.47 to 17.03) |
| **2011** | 28525 | 12.99 (12.84 to 13.15) | 14250 | 10.60 (10.42 to 10.78) | 14275 | 16.32 (16.05 to 16.59) |
| **2012** | 28149 | 12.53 (12.38 to 12.67) | 14099 | 10.27 (10.10 to 10.44) | 14050 | 15.58 (15.32 to 15.84) |
| **2013** | 27338 | 11.90 (11.75 to 12.04) | 13221 | 9.48 (9.31 to 9.64) | 14117 | 15.13 (14.87 to 15.38) |
| **2014** | 26125 | 11.07 (10.93 to 11.20) | 12601 | 8.86 (8.71 to 9.02) | 13524 | 14.02 (13.78 to 14.26) |
| **2015** | 26525 | 10.99 (10.86 to 11.12) | 12532 | 8.66 (8.51 to 8.82) | 13993 | 14.05 (13.82 to 14.29) |
| **2016** | 25969 | 10.52 (10.39 to 10.65) | 12024 | 8.19 (8.04 to 8.34) | 13945 | 13.70 (13.46 to 13.93) |
| **2017** | 26674 | 10.57 (10.44 to 10.70) | 12173 | 8.11 (7.96 to 8.26) | 14501 | 13.82 (13.59 to 14.05) |
| **2018** | 26739 | 10.33 (10.20 to 10.45) | 12004 | 7.88 (7.74 to 8.02) | 14735 | 13.61 (13.39 to 13.84) |
| **2019** | 27168 | 10.29 (10.17 to 10.42) | 12231 | 7.87 (7.73 to 8.01) | 14937 | 13.40 (13.18 to 13.62) |
| **2020** | 31286 | 11.61 (11.48 to 11.74) | 13567 | 8.63 (8.48 to 8.78) | 17719 | 15.52 (15.29 to 15.75) |
| **2021** | 30872 | 12.03 (11.89 to 12.17) | 13467 | 9.10 (8.95 to 9.26) | 17405 | 15.77 (15.54 to 16.02) |
| **2022** | 31405 | 11.56 (11.43 to 11.69) | 13502 | 8.61 (8.46 to 8.76) | 17903 | 15.52 (15.29 to 15.75) |
| **2023** | 30045 | 11.04 (10.92 to 11.17) | 13041 | 8.38 (8.24 to 8.53) | 17004 | 14.48 (14.26 to 14.70) |
| **2024** | 30073 | 10.69 (10.57 to 10.81) | 12697 | 7.97 (7.83 to 8.11) | 17376 | 14.19 (13.98 to 14.41) |
| **2025** | 30680 | 10.91 (10.79 to 11.03) | 12954 | 8.10 (7.96 to 8.24) | 17726 | 14.46 (14.24 to 14.68) |
| **Total/Average** | 876383 | 15.01 (14.85 to 15.17) | 437257 | 12.37 (12.19 to 12.56) | 439126 | 18.61 (18.32 to 18.90) |

**Supplementary Table S2.** Trends in Age-Adjusted Mortality Rates and Deaths Involving Coexisting ASCVD- and Stroke-Related Conditions Among United States Adults Aged ≥25 Years, Stratified by Age Group, 1999–2025.

| **Year** | **25-44 Years** | | **45-64 Years** | | **65-85+ Years** | |
| --- | --- | --- | --- | --- | --- | --- |
|  | **Deaths** | **AAMR (95% CI)** | **Deaths** | **AAMR (95% CI)** | **Deaths** | **AAMR (95% CI)** |
| **1999** | 185 | 0.26 (0.23-0.3) | 2925 | 4.83 (4.65-5) | 43970 | 128.1 (126.91-129.3) |
| **2000** | 165 | 0.21 (0.18-0.24) | 2879 | 4.67 (4.5-4.84) | 43385 | 124.81 (123.64-125.98) |
| **2001** | 160 | 0.21 (0.18-0.24) | 2811 | 4.41 (4.25-4.57) | 40954 | 116.05 (114.93-117.18) |
| **2002** | 160 | 0.21 (0.18-0.24) | 2792 | 4.14 (3.98-4.29) | 39949 | 111.95 (110.85-113.05) |
| **2003** | 183 | 0.26 (0.23-0.3) | 2891 | 4.12 (3.97-4.27) | 37520 | 103.62 (102.57-104.66) |
| **2004** | 161 | 0.21 (0.18-0.24) | 2753 | 3.76 (3.62-3.9) | 34561 | 94.31 (93.31-95.3) |
| **2005** | 168 | 0.21 (0.18-0.24) | 2826 | 3.72 (3.58-3.86) | 32680 | 87.54 (86.6-88.49) |
| **2006** | 165 | 0.21 (0.18-0.24) | 2840 | 3.64 (3.51-3.78) | 30676 | 80.63 (79.73-81.53) |
| **2007** | 163 | 0.21 (0.18-0.24) | 2668 | 3.31 (3.18-3.43) | 29258 | 75.35 (74.48-76.21) |
| **2008** | 179 | 0.26 (0.22-0.3) | 2625 | 3.17 (3.05-3.29) | 28113 | 71 (70.17-71.83) |
| **2009** | 166 | 0.21 (0.18-0.24) | 2750 | 3.25 (3.13-3.37) | 26384 | 65.28 (64.49-66.07) |
| **2010** | 163 | 0.21 (0.18-0.24) | 2645 | 3.03 (2.92-3.15) | 25937 | 63.2 (62.43-63.97) |
| **2011** | 148 | 0.21 (0.18-0.24) | 2741 | 3.03 (2.92-3.15) | 25636 | 60.67 (59.92-61.41) |
| **2012** | 153 | 0.21 (0.18-0.24) | 2694 | 2.99 (2.88-3.11) | 25302 | 58.34 (57.62-59.07) |
| **2013** | 169 | 0.26 (0.22-0.3) | 2794 | 3.05 (2.94-3.17) | 24375 | 54.88 (54.18-55.57) |
| **2014** | 165 | 0.22 (0.18-0.25) | 2727 | 2.93 (2.82-3.04) | 23233 | 50.96 (50.3-51.63) |
| **2015** | 150 | 0.21 (0.18-0.24) | 2856 | 3.03 (2.92-3.15) | 23519 | 50.41 (49.76-51.06) |
| **2016** | 183 | 0.22 (0.18-0.25) | 2754 | 2.89 (2.78-3) | 23032 | 48.23 (47.61-48.86) |
| **2017** | 192 | 0.26 (0.23-0.3) | 2859 | 2.99 (2.88-3.1) | 23623 | 48.21 (47.59-48.84) |
| **2018** | 166 | 0.21 (0.18-0.24) | 3026 | 3.17 (3.06-3.29) | 23547 | 46.78 (46.17-47.38) |
| **2019** | 188 | 0.26 (0.23-0.3) | 3076 | 3.21 (3.09-3.33) | 23904 | 46.41 (45.81-47) |
| **2020** | 201 | 0.26 (0.23-0.3) | 3606 | 3.79 (3.66-3.91) | 27479 | 52.15 (51.53-52.77) |
| **2021** | 239 | 0.32 (0.28-0.36) | 3719 | 3.99 (3.86-4.12) | 26914 | 53.79 (53.15-54.44) |
| **2022** | 276 | 0.32 (0.28-0.36) | 3657 | 3.9 (3.78-4.04) | 27472 | 51.53 (50.92-52.15) |
| **2023** | 267 | 0.32 (0.28-0.36) | 3511 | 3.79 (3.66-3.92) | 26267 | 49.11 (48.51-49.71) |
| **2024** | 279 | 0.32 (0.28-0.36) | 3600 | 3.91 (3.78-4.04) | 26194 | 47.06 (46.49-47.64) |
| **2025** | 264 | 0.32 (0.28-0.36) | 3538 | 3.87 (3.74-4) | 26878 | 48.27 (47.69-48.85) |
| **Total/Average** | 5058 | 0.24 (0.21 to 0.28) | 80563 | 3.58 (3.45 to 3.71) | 790762 | 69.95 (69.16 to 70.74) |

**Supplementary Table S3.** Trends in Age-Adjusted Mortality Rates and Deaths Involving Coexisting ASCVD- and Stroke-Related Conditions Among United States Adults Aged ≥25 Years, Stratified by Race and Ethnicity, 1999–2025.

| **Year** | **NH American Indian or Alaska Native** | | **NH Asian or Pacific Islander** | | **NH Black or African American** | | **NH White** | | **Hispanic or Latino** | |
| --- | --- | --- | --- | --- | --- | --- | --- | --- | --- | --- |
|  | **Deaths** | **AAMR (95% CI)** | **Deaths** | **AAMR (95% CI)** | **Deaths** | **AAMR (95% CI)** | **Deaths** | **AAMR (95% CI)** | **Deaths** | **AAMR (95% CI)** |
| **1999** | 125 | 20.2 (16.52 - 23.88) | 821 | 23.78 (22.1 - 25.47) | 4532 | 32.23 (31.28 - 33.17) | 39805 | 26.42 (26.16 - 26.68) | 1797 | 23.9 (22.76 - 25.04) |
| **2000** | 141 | 20.45 (16.95 - 23.94) | 764 | 20.89 (19.36 - 22.42) | 4559 | 31.97 (31.03 - 32.9) | 39165 | 25.73 (25.48 - 25.99) | 1800 | 23.02 (21.93 - 24.12) |
| **2001** | 130 | 18.49 (15.19 - 21.8) | 778 | 19.17 (17.78 - 20.56) | 4454 | 30.76 (29.85 - 31.68) | 36757 | 23.86 (23.62 - 24.11) | 1806 | 21.52 (20.5 - 22.55) |
| **2002** | 121 | 16.45 (13.4 - 19.5) | 834 | 19.35 (18 - 20.7) | 4411 | 30.21 (29.31 - 31.11) | 35759 | 22.98 (22.75 - 23.22) | 1776 | 20.24 (19.27 - 21.21) |
| **2003** | 150 | 20.39 (17 - 23.77) | 845 | 18.49 (17.2 - 19.77) | 4227 | 28.44 (27.57 - 29.31) | 33581 | 21.3 (21.08 - 21.53) | 1791 | 19.09 (18.18 - 20) |
| **2004** | 143 | 18.61 (15.41 - 21.8) | 768 | 15.86 (14.71 - 17.01) | 3935 | 26.05 (25.22 - 26.88) | 30880 | 19.39 (19.18 - 19.61) | 1749 | 17.83 (16.97 - 18.69) |
| **2005** | 136 | 16.18 (13.31 - 19.04) | 819 | 15.51 (14.42 - 16.59) | 3694 | 23.63 (22.86 - 24.4) | 29282 | 18.11 (17.91 - 18.32) | 1743 | 16.75 (15.95 - 17.56) |
| **2006** | 130 | 15.71 (12.87 - 18.55) | 799 | 14.42 (13.4 - 15.45) | 3492 | 21.7 (20.97 - 22.43) | 27554 | 16.76 (16.56 - 16.96) | 1706 | 15.41 (14.66 - 16.16) |
| **2007** | 121 | 14.31 (11.64 - 16.97) | 741 | 12.41 (11.5 - 13.33) | 3510 | 21.24 (20.52 - 21.95) | 26066 | 15.61 (15.42 - 15.8) | 1651 | 14.28 (13.57 - 14.99) |
| **2008** | 116 | 12.91 (10.43 - 15.39) | 737 | 11.71 (10.85 - 12.58) | 3478 | 20.59 (19.89 - 21.29) | 25063 | 14.77 (14.59 - 14.96) | 1523 | 12.24 (11.6 - 12.87) |
| **2009** | 118 | 13.06 (10.59 - 15.53) | 711 | 10.66 (9.86 - 11.46) | 3171 | 18.1 (17.46 - 18.75) | 23764 | 13.79 (13.62 - 13.97) | 1536 | 11.69 (11.09 - 12.29) |
| **2010** | 119 | 12.91 (10.48 - 15.34) | 732 | 10.56 (9.78 - 11.34) | 3045 | 16.9 (16.28 - 17.51) | 23199 | 13.26 (13.09 - 13.43) | 1650 | 12.15 (11.55 - 12.75) |
| **2011** | 141 | 14.6 (12.09 - 17.11) | 740 | 9.83 (9.11 - 10.55) | 3078 | 16.51 (15.92 - 17.11) | 22937 | 12.84 (12.68 - 13.01) | 1629 | 11.06 (10.51 - 11.61) |
| **2012** | 130 | 12.02 (9.86 - 14.17) | 764 | 9.4 (8.73 - 10.08) | 3046 | 15.72 (15.15 - 16.29) | 22566 | 12.37 (12.2 - 12.53) | 1643 | 10.55 (10.03 - 11.08) |
| **2013** | 148 | 13.35 (11.11 - 15.59) | 791 | 9.02 (8.38 - 9.65) | 3067 | 15.25 (14.7 - 15.8) | 21656 | 11.71 (11.56 - 11.87) | 1676 | 10 (9.51 - 10.49) |
| **2014** | 131 | 10.82 (8.89 - 12.75) | 755 | 7.94 (7.36 - 8.51) | 2832 | 13.4 (12.89 - 13.91) | 20708 | 10.99 (10.84 - 11.15) | 1699 | 9.49 (9.03 - 9.95) |
| **2015** | 135 | 10.61 (8.75 - 12.48) | 754 | 7.44 (6.9 - 7.98) | 3087 | 14.18 (13.67 - 14.7) | 20846 | 10.92 (10.77 - 11.07) | 1703 | 8.95 (8.52 - 9.39) |
| **2016** | 166 | 12.33 (10.38 - 14.27) | 769 | 7.19 (6.67 - 7.7) | 2958 | 13.12 (12.64 - 13.61) | 20353 | 10.54 (10.39 - 10.69) | 1723 | 8.52 (8.11 - 8.93) |
| **2017** | 146 | 10.53 (8.77 - 12.29) | 844 | 7.31 (6.81 - 7.81) | 3182 | 13.58 (13.1 - 14.06) | 20718 | 10.54 (10.39 - 10.68) | 1784 | 8.35 (7.96 - 8.75) |
| **2018** | 172 | 11.5 (9.73 - 13.27) | 852 | 7.02 (6.54 - 7.5) | 3190 | 13.19 (12.73 - 13.66) | 20723 | 10.33 (10.18 - 10.47) | 1802 | 8.11 (7.73 - 8.49) |
| **2019** | 149 | 9.18 (7.66 - 10.7) | 894 | 6.94 (6.48 - 7.4) | 3198 | 12.76 (12.31 - 13.22) | 21081 | 10.35 (10.21 - 10.49) | 1846 | 7.97 (7.59 - 8.34) |
| **2020** | 196 | 11.74 (10.05 - 13.43) | 1103 | 8.22 (7.73 - 8.71) | 4006 | 15.43 (14.94 - 15.92) | 23647 | 11.51 (11.36 - 11.66) | 2334 | 9.55 (9.15 - 9.94) |
| **2021** | 178 | 11.71 (10 - 13.63) | 1078 | 8.19 (7.71 - 8.71) | 3829 | 15.17 (14.68 - 15.68) | 23610 | 12.17 (12.01 - 12.33) | 2177 | 9.01 (8.62 - 9.41) |
| **2022** | 200 | 12.26 (10.59 - 14.15) | 980 | 6.92 (6.49 - 7.37) | 3943 | 15.18 (14.69 - 15.67) | 24057 | 11.74 (11.59 - 11.89) | 2225 | 8.7 (8.33 - 9.08) |
| **2023** | 201 | 11.76 (10.16 - 13.56) | 1018 | 6.93 (6.51 - 7.38) | 3799 | 14.29 (13.83 - 14.76) | 22824 | 11.19 (11.04 - 11.34) | 2203 | 8.23 (7.88 - 8.59) |
| **2024** | 172 | 9.62 (8.21 - 11.22) | 1047 | 6.61 (6.21 - 7.02) | 3759 | 13.45 (13.01 - 13.9) | 22826 | 10.94 (10.8 - 11.09) | 2269 | 7.97 (7.63 - 8.31) |
| **2025** | 174 | 9.66 (8.25 - 11.25) | 1096 | 6.91 (6.5 - 7.33) | 3907 | 13.96 (13.52 - 14.42) | 23197 | 11.12 (10.98 - 11.27) | 2306 | 8.06 (7.72 - 8.41) |
| **Total/Average** | 3989 | 13.75 (11.42 to 16.13) | 22834 | 11.43 (10.63 to 12.24) | 97389 | 19.15 (18.52 to 19.78) | 702624 | 14.86 (14.68 to 15.04) | 49547 | 12.69 (12.09 to 13.30) |

**Supplementary Table S4. Annual Death Counts for Small-Denominator Subgroups: Adults Aged 25–44 Years and NH American Indian or Alaska Native Adults, 1999–2025.**

| **Year** | **25–44 Years Deaths** | **AAMR (95% CI)** | **NH AIAN Deaths** | **AAMR (95% CI)** |
| --- | --- | --- | --- | --- |
| 1999 | 185 | 0.26 (0.23–0.30) | 125 | 20.20 (16.52–23.88) |
| 2000 | 165 | 0.21 (0.18–0.24) | 141 | 20.45 (16.95–23.94) |
| 2001 | 160 | 0.21 (0.18–0.24) | 130 | 18.49 (15.19–21.80) |
| 2002 | 160 | 0.21 (0.18–0.24) | 121 | 16.45 (13.40–19.50) |
| 2003 | 183 | 0.26 (0.23–0.30) | 150 | 20.39 (17.00–23.77) |
| 2004 | 161 | 0.21 (0.18–0.24) | 143 | 18.61 (15.41–21.80) |
| 2005 | 168 | 0.21 (0.18–0.24) | 136 | 16.18 (13.31–19.04) |
| 2006 | 165 | 0.21 (0.18–0.24) | 130 | 15.71 (12.87–18.55) |
| 2007 | 163 | 0.21 (0.18–0.24) | 121 | 14.31 (11.64–16.97) |
| 2008 | 179 | 0.26 (0.22–0.30) | 116 | 12.91 (10.43–15.39) |
| 2009 | 166 | 0.21 (0.18–0.24) | 118 | 13.06 (10.59–15.53) |
| 2010 | 163 | 0.21 (0.18–0.24) | 119 | 12.91 (10.48–15.34) |
| 2011 | 148 | 0.21 (0.18–0.24) | 141 | 14.60 (12.09–17.11) |
| 2012 | 153 | 0.21 (0.18–0.24) | 130 | 12.02 (9.86–14.17) |
| 2013 | 169 | 0.26 (0.22–0.30) | 148 | 13.35 (11.11–15.59) |
| 2014 | 165 | 0.22 (0.18–0.25) | 131 | 10.82 (8.89–12.75) |
| 2015 | 150 | 0.21 (0.18–0.24) | 135 | 10.61 (8.75–12.48) |
| 2016 | 183 | 0.22 (0.18–0.25) | 166 | 12.33 (10.38–14.27) |
| 2017 | 192 | 0.26 (0.23–0.30) | 146 | 10.53 (8.77–12.29) |
| 2018 | 166 | 0.21 (0.18–0.24) | 172 | 11.50 (9.73–13.27) |
| 2019 | 188 | 0.26 (0.23–0.30) | 149 | 9.18 (7.66–10.70) |
| 2020 | 201 | 0.26 (0.23–0.30) | 196 | 11.74 (10.05–13.43) |
| 2021 | 239 | 0.32 (0.28–0.36) | 178 | 11.71 (10.00–13.63) |
| 2022 | 276 | 0.32 (0.28–0.36) | 200 | 12.26 (10.59–14.15) |
| 2023 | 267 | 0.32 (0.28–0.36) | 201 | 11.76 (10.16–13.56) |
| 2024 | 279 | 0.32 (0.28–0.36) | 172 | 9.62 (8.21–11.22) |
| 2025 | 264 | 0.32 (0.28–0.36) | 174 | 9.66 (8.25–11.25) |
| **Total** | **5,058** | **0.24 (0.21–0.28)** | **3,989** | **13.75 (11.42–16.13)** |

**Footnote:** Annual death counts are reported to allow assessment of CDC WONDER data reliability thresholds. CDC WONDER suppresses counts below 10 and flags rates as unreliable when annual deaths fall below 20. All annual counts in both subgroups exceed 100 throughout the study period, confirming that no suppression or reliability flagging occurred and that Joinpoint trend estimates for these subgroups rest on stable annual numerators. Wide confidence intervals around NH American Indian or Alaska Native AAMR estimates reflect population size rather than count instability.

**Supplementary Table S5.** Trends in Age-Adjusted Mortality Rates and Deaths Involving Coexisting ASCVD- and Stroke-Related Conditions Among United States Adults Aged ≥25 Years, Stratified by Urbanization, 1999–2020.

| **Year** | **Metropolitan** | | **Non-metropolitan** | |
| --- | --- | --- | --- | --- |
|  | **Deaths** | **AAMR (95% CI)** | **Deaths** | **AAMR (95% CI)** |
| **1999** | 37144 | 26.22 (25.96 - 26.49) | 9936 | 29.16 (28.59 - 29.74) |
| **2000** | 36601 | 25.49 (25.23 - 25.75) | 9828 | 28.6 (28.03 - 29.17) |
| **2001** | 34664 | 23.66 (23.41 - 23.91) | 9261 | 26.77 (26.23 - 27.32) |
| **2002** | 33769 | 22.72 (22.48 - 22.96) | 9132 | 26.26 (25.72 - 26.8) |
| **2003** | 31826 | 21 (20.77 - 21.24) | 8768 | 25.05 (24.52 - 25.57) |
| **2004** | 29452 | 19.16 (18.94 - 19.38) | 8023 | 22.69 (22.19 - 23.18) |
| **2005** | 27994 | 17.79 (17.58 - 18) | 7680 | 21.53 (21.04 - 22.01) |
| **2006** | 26491 | 16.47 (16.27 - 16.67) | 7190 | 19.83 (19.37 - 20.28) |
| **2007** | 25240 | 15.35 (15.16 - 15.54) | 6849 | 18.63 (18.19 - 19.07) |
| **2008** | 24225 | 14.41 (14.23 - 14.59) | 6692 | 18.01 (17.57 - 18.44) |
| **2009** | 22994 | 13.36 (13.18 - 13.53) | 6306 | 16.76 (16.35 - 17.18) |
| **2010** | 22620 | 12.93 (12.76 - 13.1) | 6125 | 16.07 (15.66 - 16.47) |
| **2011** | 22476 | 12.46 (12.29 - 12.62) | 6049 | 15.61 (15.21 - 16) |
| **2012** | 22193 | 11.96 (11.81 - 12.12) | 5956 | 15.09 (14.7 - 15.47) |
| **2013** | 21697 | 11.39 (11.24 - 11.55) | 5641 | 14.09 (13.72 - 14.46) |
| **2014** | 20707 | 10.57 (10.42 - 10.71) | 5418 | 13.32 (12.96 - 13.68) |
| **2015** | 21046 | 10.51 (10.37 - 10.65) | 5479 | 13.3 (12.94 - 13.65) |
| **2016** | 20517 | 10.03 (9.89 - 10.17) | 5452 | 13.09 (12.74 - 13.44) |
| **2017** | 21140 | 10.07 (9.93 - 10.2) | 5534 | 13.07 (12.72 - 13.41) |
| **2018** | 21139 | 9.78 (9.65 - 9.92) | 5600 | 12.9 (12.56 - 13.25) |
| **2019** | 21275 | 9.66 (9.53 - 9.79) | 5893 | 13.4 (13.05 - 13.74) |
| **2020** | 24752 | 11.02 (10.88 - 11.16) | 6534 | 14.66 (14.3 - 15.02) |
| **Total/Average** | 569962 | 15.27 (15.09 to 15.46) | 153346 | 18.54 (18.11 to 18.97) |

**Supplementary Table S6.** Trends in Age-Adjusted Mortality Rates and Deaths Involving Coexisting ASCVD- and Stroke-Related Conditions Among United States Adults Aged ≥25 Years, Stratified by Census Region, 1999–2025.

| **Year** | **Northeast** | | **Midwest** | | **South** | | **West** | |
| --- | --- | --- | --- | --- | --- | --- | --- | --- |
|  | **Deaths** | **AAMR (95% CI)** | **Deaths** | **AAMR (95% CI)** | **Deaths** | **AAMR (95% CI)** | **Deaths** | **AAMR (95% CI)** |
| **1999** | 9428 | 24.94 (24.43 - 25.44) | 11540 | 27.03 (26.54 - 27.53) | 16265 | 26.65 (26.24 - 27.06) | 9847 | 28.78 (28.21 - 29.35) |
| **2000** | 9355 | 24.41 (23.91 - 24.9) | 11103 | 25.79 (25.31 - 26.27) | 16320 | 26.37 (25.97 - 26.78) | 9651 | 27.67 (27.12 - 28.23) |
| **2001** | 8714 | 22.44 (21.96 - 22.91) | 10505 | 24.12 (23.66 - 24.58) | 15439 | 24.6 (24.21 - 24.99) | 9267 | 25.87 (25.34 - 26.4) |
| **2002** | 8336 | 21.21 (20.75 - 21.66) | 10191 | 23.17 (22.72 - 23.62) | 15168 | 23.85 (23.47 - 24.23) | 9206 | 25.17 (24.66 - 25.69) |
| **2003** | 7879 | 19.74 (19.3 - 20.17) | 9509 | 21.33 (20.9 - 21.76) | 14306 | 22.14 (21.78 - 22.51) | 8900 | 23.72 (23.23 - 24.21) |
| **2004** | 7104 | 17.66 (17.25 - 18.07) | 8871 | 19.72 (19.31 - 20.13) | 13249 | 20.11 (19.77 - 20.45) | 8251 | 21.56 (21.1 - 22.03) |
| **2005** | 6644 | 16.3 (15.91 - 16.69) | 8612 | 18.89 (18.49 - 19.29) | 12832 | 19.05 (18.72 - 19.38) | 7586 | 19.26 (18.83 - 19.7) |
| **2006** | 6061 | 14.63 (14.26 - 15.0) | 8002 | 17.28 (16.9 - 17.66) | 12139 | 17.56 (17.25 - 17.88) | 7479 | 18.45 (18.04 - 18.87) |
| **2007** | 5956 | 14.21 (13.84 - 14.57) | 7709 | 16.33 (15.96 - 16.69) | 11568 | 16.36 (16.06 - 16.66) | 6856 | 16.47 (16.08 - 16.86) |
| **2008** | 5632 | 13.19 (12.85 - 13.54) | 7455 | 15.58 (15.23 - 15.94) | 11099 | 15.32 (15.03 - 15.6) | 6731 | 15.74 (15.36 - 16.12) |
| **2009** | 5326 | 12.31 (11.97 - 12.64) | 6925 | 14.27 (13.94 - 14.61) | 10723 | 14.45 (14.18 - 14.73) | 6326 | 14.34 (13.98 - 14.7) |
| **2010** | 5224 | 11.95 (11.63 - 12.28) | 6779 | 13.77 (13.44 - 14.11) | 10584 | 13.99 (13.72 - 14.26) | 6158 | 13.75 (13.4 - 14.09) |
| **2011** | 5137 | 11.59 (11.27 - 11.91) | 6616 | 13.13 (12.81 - 13.44) | 10465 | 13.34 (13.08 - 13.6) | 6307 | 13.52 (13.18 - 13.86) |
| **2012** | 5048 | 11.13 (10.82 - 11.44) | 6366 | 12.42 (12.12 - 12.73) | 10663 | 13.21 (12.96 - 13.46) | 6072 | 12.61 (12.29 - 12.93) |
| **2013** | 4781 | 10.4 (10.1 - 10.7) | 6251 | 12.01 (11.7 - 12.31) | 10248 | 12.3 (12.06 - 12.54) | 6058 | 12.2 (11.89 - 12.51) |
| **2014** | 4589 | 9.89 (9.6 - 10.18) | 6035 | 11.37 (11.08 - 11.66) | 9593 | 11.2 (10.98 - 11.43) | 5908 | 11.53 (11.23 - 11.83) |
| **2015** | 4511 | 9.57 (9.28 - 9.85) | 5954 | 11.09 (10.8 - 11.37) | 10037 | 11.37 (11.15 - 11.59) | 6023 | 11.35 (11.06 - 11.64) |
| **2016** | 4274 | 9.0 (8.73 - 9.27) | 5751 | 10.57 (10.29 - 10.85) | 10106 | 11.16 (10.94 - 11.38) | 5838 | 10.72 (10.44 - 10.99) |
| **2017** | 4328 | 8.83 (8.56 - 9.09) | 5983 | 10.81 (10.54 - 11.09) | 10332 | 11.09 (10.87 - 11.3) | 6031 | 10.85 (10.58 - 11.13) |
| **2018** | 4348 | 8.76 (8.49 - 9.02) | 5992 | 10.64 (10.37 - 10.91) | 10640 | 11.08 (10.87 - 11.3) | 5759 | 10.04 (9.78 - 10.3) |
| **2019** | 4339 | 8.64 (8.38 - 8.9) | 6021 | 10.49 (10.22 - 10.75) | 11017 | 11.18 (10.97 - 11.39) | 5791 | 9.88 (9.62 - 10.13) |
| **2020** | 4983 | 9.87 (9.59 - 10.15) | 7020 | 12.13 (11.84 - 12.41) | 12647 | 12.5 (12.28 - 12.72) | 6636 | 11.04 (10.77 - 11.31) |
| **2021** | 4782 | 9.78 (9.5 - 10.06) | 6647 | 12.14 (11.85 - 12.44) | 12856 | 13.26 (13.03 - 13.5) | 6587 | 11.63 (11.34 - 11.91) |
| **2022** | 4649 | 9.07 (8.81 - 9.34) | 6780 | 11.75 (11.47 - 12.04) | 13150 | 12.85 (12.63 - 13.07) | 6826 | 11.3 (11.03 - 11.58) |
| **2023** | 4416 | 8.62 (8.37 - 8.89) | 6406 | 11.17 (10.9 - 11.45) | 12857 | 12.45 (12.24 - 12.67) | 6366 | 10.52 (10.26 - 10.78) |
| **2024** | 4243 | 8.06 (7.82 - 8.31) | 6659 | 11.31 (11.03 - 11.59) | 12757 | 11.92 (11.71 - 12.13) | 6414 | 10.23 (9.98 - 10.48) |
| **2025** | 4446 | 8.4 (8.15 - 8.66) | 6560 | 11.12 (10.85 - 11.4) | 13124 | 12.25 (12.04 - 12.46) | 6550 | 10.43 (10.18 - 10.69) |
| **Total/Average** | 154533 | 13.13 (12.80 - 13.47) | 202242 | 15.16 (14.82 - 15.50) | 330184 | 15.62 (15.34 - 15.89) | 189424 | 15.50 (15.15 - 15.86) |

**Supplementary Table S7.** Percentage Distribution of Place of Death Among United States Adults Aged ≥25 Years With Mortality Involving Coexisting ASCVD- and Stroke-Related Conditions, 1999–2025.

| **Place of Death** | **Deaths** | **% Total** |
| --- | --- | --- |
| **Medical Facility - Inpatient** | 293,299 | 33.46% |
| **Medical Facility - Outpatient or ER** | 46,431 | 5.30% |
| **Medical Facility - Dead on Arrival** | 3,664 | 0.42% |
| **Medical Facility - Status unknown** | 709 | 0.08% |
| **Decedent's home** | 182,041 | 20.77% |
| **Hospice facility** | 40,987 | 4.68% |
| **Nursing home/long term care** | 276,185 | 31.52% |
| **Other** | 31,532 | 3.60% |
| **Place of death unknown** | 1,535 | 0.18% |
| **Total** | 876383 | 100% |

**Supplementary Table S8.** Trends in Age-Adjusted Mortality Rates and Deaths Involving Coexisting ASCVD- and Stroke-Related Conditions Among United States Adults Aged ≥25 Years, Stratified by State, 1999–2025.

| **State** | **1999-2020** | | **2021-2025** | | **1999-2025** |
| --- | --- | --- | --- | --- | --- |
|  | **Deaths** | **AAMR (95% CI)** | **Deaths** | **AAMR (95% CI)** | **Deaths** |
| **Alabama** | 10725 | 14.63 (14.35 - 14.91) | 2044 | 9.24 - 10.1 | 12769 |
| **Alaska** | 696 | 12 (11.06 - 12.93) | 235 | 9.85 - 12.94 | 931 |
| **Arizona** | 10815 | 10.94 (10.73 - 11.14) | 2384 | 7.04 - 7.64 | 13199 |
| **Arkansas** | 7854 | 16.62 (16.25 - 16.99) | 1898 | 14.39 - 15.77 | 9752 |
| **California** | 93090 | 18.25 (18.13 - 18.37) | 14756 | 9.79 - 10.11 | 107846 |
| **Colorado** | 7081 | 11.16 (10.89 - 11.42) | 2741 | 13.04 - 14.07 | 9822 |
| **Connecticut** | 8419 | 13.09 (12.81 - 13.37) | 927 | 5.31 - 6.05 | 9346 |
| **Delaware** | 2429 | 16.82 (16.15 - 17.5) | 610 | 12.03 - 14.19 | 3039 |
| **District of Columbia** | 1843 | 21.69 (20.69 - 22.69) | 317 | 13.03 - 16.33 | 2160 |
| **Florida** | 45654 | 11.87 (11.76 - 11.98) | 11682 | 9.8 - 10.17 | 57336 |
| **Georgia** | 13513 | 11.66 (11.46 - 11.85) | 3232 | 8.09 - 8.68 | 16745 |
| **Hawaii** | 3005 | 12.77 (12.31 - 13.24) | 477 | 6.43 - 7.78 | 3482 |
| **Idaho** | 2686 | 12.21 (11.75 - 12.68) | 887 | 11.42 - 13.06 | 3573 |
| **Illinois** | 25308 | 13.02 (12.86 - 13.19) | 3627 | 6.77 - 7.23 | 28935 |
| **Indiana** | 16082 | 16.3 (16.05 - 16.55) | 3460 | 12.34 - 13.21 | 19542 |
| **Iowa** | 9337 | 16.17 (15.84 - 16.5) | 1873 | 12.58 - 13.8 | 11210 |
| **Kansas** | 5673 | 12.04 (11.72 - 12.35) | 1184 | 9.42 - 10.59 | 6857 |
| **Kentucky** | 11177 | 17.25 (16.93 - 17.58) | 2449 | 12.9 - 13.99 | 13626 |
| **Louisiana** | 8801 | 13.56 (13.28 - 13.85) | 2865 | 15.39 - 16.58 | 11666 |
| **Maine** | 3450 | 13.97 (13.5 - 14.44) | 644 | 8.34 - 9.79 | 4094 |
| **Maryland** | 15069 | 18.01 (17.73 - 18.3) | 3660 | 14.15 - 15.11 | 18729 |
| **Massachusetts** | 10004 | 8.79 (8.62 - 8.97) | 1915 | 6.05 - 6.63 | 11919 |
| **Michigan** | 24982 | 15.57 (15.38 - 15.77) | 4881 | 11.14 - 11.79 | 29863 |
| **Minnesota** | 12462 | 14.64 (14.38 - 14.9) | 3982 | 16.2 - 17.26 | 16444 |
| **Mississippi** | 9592 | 22.22 (21.78 - 22.67) | 2557 | 20.78 - 22.49 | 12149 |
| **Missouri** | 13685 | 13.94 (13.71 - 14.18) | 2369 | 8.68 - 9.43 | 16054 |
| **Montana** | 1952 | 11.41 (10.9 - 11.92) | 630 | 11.66 - 13.71 | 2582 |
| **Nebraska** | 4906 | 15.84 (15.4 - 16.29) | 1096 | 13 - 14.67 | 6002 |
| **Nevada** | 2748 | 8 (7.7 - 8.3) | 1211 | 9.56 - 10.73 | 3959 |
| **New Hampshire** | 2680 | 12.67 (12.19 - 13.15) | 587 | 8.32 - 9.83 | 3267 |
| **New Jersey** | 21568 | 14.91 (14.71 - 15.11) | 3456 | 8.47 - 9.06 | 25024 |
| **New Mexico** | 3718 | 12.38 (11.98 - 12.78) | 954 | 9.74 - 11.1 | 4672 |
| **New York** | 40562 | 12.63 (12.5 - 12.75) | 7643 | 8.5 - 8.9 | 48205 |
| **North Carolina** | 22610 | 16.32 (16.11 - 16.54) | 5091 | 11.54 - 12.21 | 27701 |
| **North Dakota** | 2140 | 16.71 (15.99 - 17.43) | 390 | 10.85 - 13.34 | 2530 |
| **Ohio** | 39648 | 20.61 (20.4 - 20.81) | 5978 | 11.61 - 12.22 | 45626 |
| **Oklahoma** | 12937 | 22.52 (22.13 - 22.91) | 3339 | 21.16 - 22.68 | 16276 |
| **Oregon** | 9578 | 15.12 (14.82 - 15.43) | 2926 | 15.7 - 16.9 | 12504 |
| **Pennsylvania** | 39104 | 16.14 (15.98 - 16.31) | 6257 | 9.98 - 10.5 | 45361 |
| **Rhode Island** | 3887 | 19.58 (18.96 - 20.21) | 605 | 11.1 - 13.08 | 4492 |
| **South Carolina** | 11139 | 16.04 (15.74 - 16.34) | 3190 | 13.65 - 14.66 | 14329 |
| **South Dakota** | 2465 | 16.54 (15.88 - 17.2) | 540 | 13.09 - 15.58 | 3005 |
| **Tennessee** | 20190 | 21.37 (21.07 - 21.67) | 4210 | 14.62 - 15.55 | 24400 |
| **Texas** | 50708 | 17.07 (16.92 - 17.21) | 12664 | 13.12 - 13.59 | 63372 |
| **Utah** | 2531 | 8.97 (8.62 - 9.32) | 685 | 6.98 - 8.13 | 3216 |
| **Vermont** | 2323 | 21.62 (20.74 - 22.51) | 502 | 14.89 - 17.84 | 2825 |
| **Virginia** | 13112 | 11.78 (11.58 - 11.98) | 3489 | 9.8 - 10.49 | 16601 |
| **Washington** | 17762 | 18.14 (17.88 - 18.41) | 4522 | 14.91 - 15.82 | 22284 |
| **West Virginia** | 8087 | 24.26 (23.73 - 24.79) | 1447 | 16.14 - 17.95 | 9534 |
| **Wisconsin** | 12502 | 13.14 (12.91 - 13.37) | 3672 | 14.01 - 14.96 | 16174 |
| **Wyoming** | 1019 | 12.84 (12.05 - 13.63) | 335 | 12.72 - 15.88 | 1354 |

**Supplementary Table S9. Sensitivity Analyses of Mortality Involving Coexisting ASCVD- and Stroke-Related Conditions Using Alternative Death-Certificate Definitions.**

**Panel A: Alternative Cause-of-Death Classification Definitions**

| **Definition** | **AAMR 1999 (95% CI)** | **AAMR 2025 (95% CI)** | **APC Late-Period Surge Segment (95% CI)** | **APC Post-Surge Segment (95% CI)** | **AAPC 1999–2025 (95% CI)** |
| --- | --- | --- | --- | --- | --- |
| UCD ASCVD | 186.79 (186.15–187.43) | 91.73 (91.37–92.09) | 2018–2021: 1.55 (−1.50 to 4.69) | 2021–2025: −3.34 (−4.27 to −2.41) | −2.75 (−3.15 to −2.36) |
| UCD Stroke | 81.42 (81.00–81.84) | 38.78 (38.55–39.02) | 2018–2021: 0.96 (−2.65 to 4.71) | 2021–2025: −2.91 (−4.00 to −1.80) | −2.90 (−3.42 to −2.37) |
| UCD ASCVD with Stroke | 13.90 (13.72–14.07) | 4.12 (4.04–4.19) | 2009–2016: −4.89 (−6.13 to −3.63) | 2016–2025: −0.94 (−1.71 to −0.17) | −4.58 (−5.15 to −4.01) |
| UCD Stroke with ASCVD | 5.71 (5.60–5.82) | 2.66 (2.60–2.73) | 2018–2021: 5.59 (−3.65 to 15.70) | 2021–2025: −1.73 (−4.31 to 0.93) | −3.13 (−4.20 to −2.05) |

**Panel B: ICD-10 Code Restriction and Provisional Data Sensitivity Analyses**

| Definition | Total Deaths | AAMR 1999 (95% CI) | AAMR Terminal Year (95% CI) | 2018–2021 APC (95% CI) | p-value | AAPC (95% CI) |
| --- | --- | --- | --- | --- | --- | --- |
| Primary analysis: broad ASCVD codes + stroke (I60, I61, I63, I64, I69.0, I69.1, I69.3, I69.4), 1999–2025 | 876,383 | 26.82 (26.57–27.06) | 10.91 (10.79–11.03) [2025] | 6.06 (0.01 to 12.48) | 0.050 | −3.53 (−4.27 to −2.78) |
| Sensitivity 1: restricted ASCVD codes (I25.x) + stroke (I60, I61, I63, I64, I69.0, I69.1, I69.3, I69.4), 1999–2025, overall | 834,046 | 26.24 (26.00–26.48) | 10.06 (9.95–10.18) [2025] | 5.74 (−0.08 to 11.90) | 0.053 | −3.75 (−4.47 to −3.03) |
| Sensitivity 2a: I25.x restriction, females, 1999–2025 | 414,982 | 23.15 (22.86–23.43) | 7.35 (7.21–7.48) [2025] | 5.10 (−0.90 to 11.46) | 0.090 | −4.44 (−5.16 to −3.72) |
| Sensitivity 2b: I25.x restriction, males, 1999–2025 | 419,064 | 30.68 (30.24–31.11) | 13.53 (13.32–13.74) [2025] | 5.94 (−1.43 to 13.87) | 0.109 | −3.32 (−4.18 to −2.45) |
| Sensitivity 3: primary broad codes (I60, I61, I63, I64, I69.0, I69.1, I69.3, I69.4), confirmed data only, 1999–2024, overall | 845,703 | 26.82 (26.57–27.06) | 10.69 (10.57–10.81) [2024] | 6.50 (1.01 to 12.30) | 0.024 | −3.66 (−4.36 to −2.95) |
| Sensitivity 3a: confirmed data only, females, 1999–2024 | 424,303 | 23.64 (23.35–23.93) | 7.97 (7.83–8.11) [2024] | 5.90 (−0.26 to 12.45) | 0.059 | −4.27 (−5.04 to −3.50) |
| Sensitivity 3b: confirmed data only, males, 1999–2024 | 421,400 | 31.29 (30.85–31.73) | 14.19 (13.98–14.41) [2024] | 6.71 (−0.17 to 14.05) | 0.055 | −3.27 (−4.12 to −2.42) |

APC = annual percent change; AAPC = average annual percent change; ASCVD = atherosclerotic cardiovascular disease; CI = confidence interval; AAMR = age-adjusted mortality rate per 100,000; UCD = underlying cause of death; I25.x = complete ICD-10 category for chronic ischemic heart disease. Stroke-related codes used throughout: I60, I61, I63, I64, I69.0, I69.1, I69.3, I69.4.

(Sensitivity 1, 2a, 2b): total deaths and AAMR values updated from the I25.x Joinpoint source file. The previously reported figure of 867,702 for Sensitivity 1 has been corrected to 834,046 (a 4.8% difference from the primary analysis). AAMR 1999 and terminal-year AAMR are now fully populated for all three I25.x-restricted strata.

(Sensitivity 3, 3a, 3b): total deaths and AAMR values populated from the confirmed 1999–2024 Joinpoint source file. Terminal year for these rows is 2024.

The 2018–2021 APC p-values are shown to allow direct comparison of the borderline primary finding across all sensitivity conditions. Under the I25.x-restricted definition, the 2018–2021 increase was non-significant across all three strata (overall p = 0.053, females p = 0.090, males p = 0.109), consistent with a modest and code-sensitive inflection. The confirmed 1999–2024 analysis yielded a stronger overall 2018–2021 signal (p = 0.024) but non-significant sex-stratified estimates, further supporting a borderline and sex-heterogeneous inflection rather than a robust acceleration.
